# Long-term analgesic effect of trans-spinal direct current stimulation compared to non-invasive motor cortex stimulation in complex regional pain syndrome

**DOI:** 10.1093/braincomms/fcad191

**Published:** 2023-07-01

**Authors:** Hasan Hodaj, Jean-Francois Payen, Enkelejda Hodaj, Marc Sorel, Anne Dumolard, Laurent Vercueil, Chantal Delon-Martin, Jean-Pascal Lefaucheur

**Affiliations:** Centre de la Douleur, Pôle Anesthésie Réanimation, CHU Grenoble Alpes, 38000 Grenoble, France; Univ. Grenoble Alpes, Inserm, U1216, Grenoble Institut Neurosciences, 38000 Grenoble, France; Centre de la Douleur, Pôle Anesthésie Réanimation, CHU Grenoble Alpes, 38000 Grenoble, France; Univ. Grenoble Alpes, Inserm, U1216, Grenoble Institut Neurosciences, 38000 Grenoble, France; Centre d'Investigation Clinique, CHU Grenoble Alpes, 38000, Grenoble, France; Centre d'Evaluation et de Traitement de la Douleur, Hôpital Sud-Seine-et-Marne, site Nemours, Nemours, France; EA 4391, Excitabilité Nerveuse et Thérapeutique, Faculté de Santé, Univ. Paris Est Créteil, Créteil, France; Centre de la Douleur, Pôle Anesthésie Réanimation, CHU Grenoble Alpes, 38000 Grenoble, France; Service de Neurologie, CHU Grenoble Alpes, 38000, Grenoble, France; Univ. Grenoble Alpes, Inserm, U1216, Grenoble Institut Neurosciences, 38000 Grenoble, France; EA 4391, Excitabilité Nerveuse et Thérapeutique, Faculté de Santé, Univ. Paris Est Créteil, Créteil, France; Unité de Neurophysiologie Clinique, Service de Physiologie—Explorations Fonctionnelles, Hôpital Henri Mondor, Assistance Publique—Hôpitaux de Paris, Créteil, France

**Keywords:** chronic pain, CRPS, rTMS, tDCS, tsDCS

## Abstract

The aim of the present study was to compare the analgesic effect of motor cortex stimulation using high-frequency repetitive transcranial magnetic stimulation or transcranial direct current stimulation and transcutaneous spinal direct current stimulation in patients with complex regional pain syndrome. Thirty-three patients with complex regional pain syndrome were randomized to one of the three treatment groups (repetitive transcranial magnetic stimulation, *n* = 11; transcranial direct current stimulation, *n* = 10; transcutaneous spinal direct current stimulation, *n* = 12) and received a series of 12 sessions of stimulation for 3 weeks (induction phase) and 11 sessions for 4 months (maintenance therapy). The primary end-point was the mean pain intensity assessed weekly with a visual numerical scale during the month prior to treatment (baseline), the 5-month stimulation period and 1 month after the treatment. The weekly visual numerical scale pain score was significantly reduced at all time points compared to baseline in the transcutaneous spinal direct current stimulation group, at the last two time points in the repetitive transcranial magnetic stimulation group (end of the 5-month stimulation period and 1 month later), but at no time point in the transcranial direct current stimulation group. A significant pain relief was observed at the end of induction phase using transcutaneous spinal direct current stimulation compared to repetitive transcranial magnetic stimulation (*P* = 0.008) and to transcranial direct current stimulation (*P* = 0.003). In this trial, transcutaneous spinal direct current stimulation was more efficient to relieve pain in patients with complex regional pain syndrome compared to motor cortex stimulation techniques (repetitive transcranial magnetic stimulation, transcranial direct current stimulation). This efficacy was found during the induction phase and was maintained thereafter. This study warrants further investigation to confirm the potentiality of transcutaneous spinal direct current stimulation as a therapeutic option in complex regional pain syndrome.

## Teaser

In a series of 33 patients with complex regional pain syndrome, Hodaj *et al*. report that transcutaneous spinal direct current stimulation is more efficient in relieving chronic pain than non-invasive stimulation of the motor cortex using repetitive transcranial magnetic stimulation or transcranial direct current stimulation.

## Graphical Abstract

**Graphical abstract:**
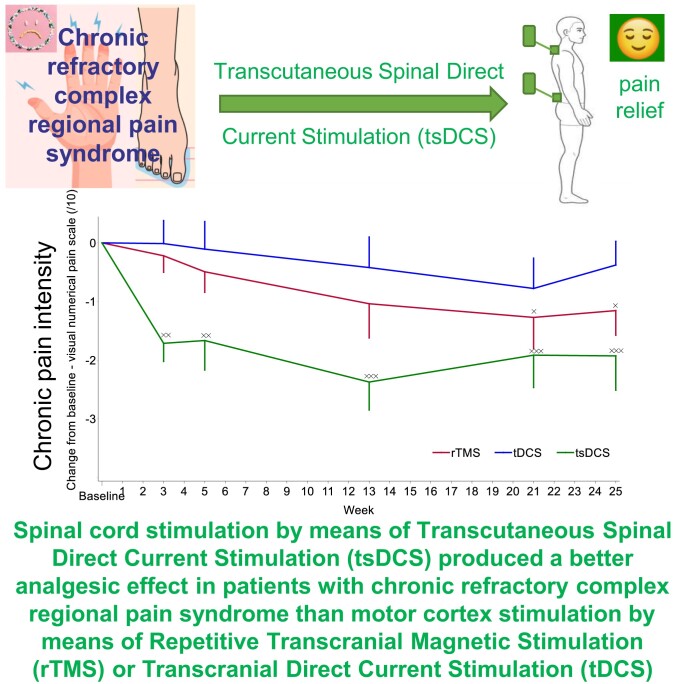

See Bocci and Priori (https://doi.org/10.1093/braincomms/fcad193) for a scientific commentary on this article.

## Introduction

Complex regional pain syndrome (CRPS) is characterized by pain and autonomic signs and symptoms usually occurring in a region of trauma or other limb injury, but disproportionate in duration or sequelae. CRPS Type I develops without evidence of nerve damage in the affected limb, through pathophysiological mechanisms that are partly unknown. However, these mechanisms likely involve maladaptive neuroplasticity in the somatosensory and autonomic (sympathetic) central nervous system.

The treatment of CRPS is based on various pharmacological or non-pharmacological approaches.^1^ Pharmacotherapy includes a wide variety of drugs, such as steroids, non-steroidal anti-inflammatory drugs, calcitonin, bisphosphonates, anticonvulsants, antidepressants, N-methyl-D-aspartate receptor antagonists or anti-hypertensives. The level of evidence depends on their use in the early or late phase of the disease. Topics can also be used, as well as locoregional ‘blocks’, in particular sympathetic nerve blocks or intravenous regional anaesthetic techniques. Non-pharmacological approaches include physiotherapy or psychotherapy, for example.

Finally, usually at the last line of the therapeutic algorithm, interventional neuromodulation, mainly based on spinal cord stimulation (SCS), can be an effective solution in chronic refractory cases.^2^ The efficacy of surgically implanted electrical motor cortex stimulation has also been reported.^3^ Following the beneficial effects obtained in the treatment of CRPS by invasive cortical neuromodulation,^4^ non-invasive techniques of cortical stimulation have been developed, such as repetitive transcranial magnetic stimulation (rTMS) and transcranial direct current stimulation (tDCS).^5^ The value of these techniques as alternatives for the treatment of refractory chronic pain has been demonstrated for 20 years.^6^

The analgesic efficacy of rTMS delivered to the motor cortex seems particularly well established. European experts have retained a level of evidence A concerning the analgesic effect of high-frequency rTMS (≥5 Hz) of the motor cortex in chronic neuropathic pain.^7^ In the French recommendations for the management of chronic neuropathic pain, rTMS is recommended as a third-line treatment.^8^ As for the use of rTMS in the treatment of chronic CRPS specifically, a recent analysis of the literature found only three controlled and short-term studies (a total of 43 patients).^9^

It is important to emphasize that rTMS requires heavy equipment with significant cost (especially if guided by neuronavigation) and the constraint for the patient to come to the hospital (clinic) to be stimulated. On the contrary, tDCS, because of its low cost and ease of use (potentially performed at home), appears in such a context as a potential and interesting alternative. Low-intensity electrical current delivered transcranially to the cerebral cortex by tDCS electrodes placed on the scalp, usually with a bipolar montage using an anode and a cathode, can modulate neural activity and induce neuroplasticity.^10^ The use of anodal tDCS delivered to the motor cortex as an analgesic therapy has provided encouraging results in patients with chronic pain.^11-13^ However, the current data in the literature do not allow to affirm, with a satisfactory level of evidence, the efficacy of tDCS in this context.^14,15^

More recently, a non-invasive neuromodulation approach to the spinal cord, transcutaneous spinal direct current stimulation (tsDCS), has been developed.^16,17^ In the somatosensory domain, studies were mainly performed in healthy subjects, showing that tsDCS is able to interfere with spinal cord structures and to modulate conduction in the lemniscal and spinothalamic sensory pathways.^18,19^ In contrast, clinical studies concerning the impact of tsDCS on pain are few. In fact, we found only two therapeutic studies reporting the analgesic effect of repeated sessions in tsDCS in series of patients suffering from various chronic pain syndromes affecting the limbs, notably related to multiple sclerosis.^20,21^ In these studies, the anode was placed over the tenth thoracic vertebra and the cathode over the right shoulder^20^ or the somatosensory cortical area.^21^

In the present study, our objective was to evaluate for the first time in CRPS patients the analgesic effect of tsDCS using a bipolar montage with both anode and cathode electrodes placed along the cervical or lumbar spine and to compare this effect to that of high-frequency rTMS and anodal tDCS delivered to the motor cortex, which are more established non-invasive neuromodulation therapies for chronic pain.

Beyond the impact of these treatments on clinical questionnaires, we also assessed the possible modulation of the autonomic nervous system by recording the sympathetic-driven electrochemical skin conductance (ESC) measured with the Sudoscan® device.

## Materials and methods

### Study design

This study was a bi-centric clinical trial randomized in three parallel arms: rTMS, tDCS and tsDCS. The study was conducted at the Pain Centre of Grenoble Alps University Hospital (Grenoble, France) and the Clinical Neurophysiology Unit of Henri Mondor University Hospital (Créteil, France) between 25 July 2016 (first visit of the first patient), and 10 September 2021 (last visit of the last patient).

**Figure 1:**
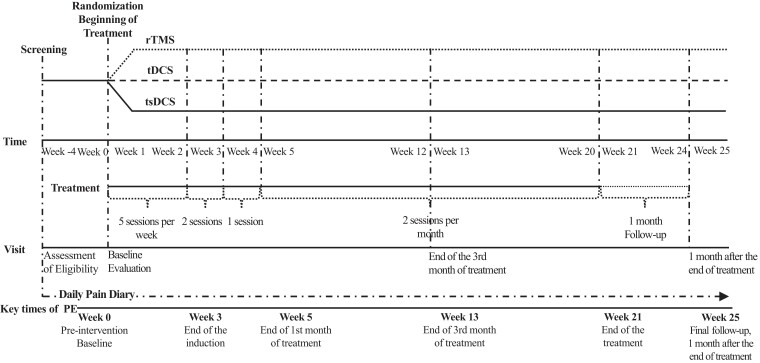
**Protocol design: interventions and assessment time points.** Assessments at each visit: patient CGI-I, SF-12; HADS, NPSI, QuickDASH, WOMAC, Sudoscan®. PE, primary end-point.

The study consisted of three periods (Fig. 1): (i) a 1-month pre-treatment period to assess eligibility and determine the average weekly level of pain intensity before treatment (Baseline); (ii) after randomization, a 5-month period of rTMS, tDCS or tsDCS sessions; and (iii) a 1-month follow-up period after treatment. Patients were evaluated at four scheduled visits: at screening to assess eligibility (Week 4), at randomization to assess baseline evaluations (Week 0), after 3 months of treatment (Week 13) and at 1-month post-treatment (Week 25).

The neuromodulation protocol of rTMS, tDCS or tsDCS consisted of an ‘induction phase’ of one stimulation session per day for 5 consecutive days during 2 weeks (Weeks 1 and 2), then 2 sessions in the next week (Week 3) for a total of 12 sessions, and a ‘maintenance phase’ consisting of one session in Week 4 and then bi-monthly sessions for 4 months, for a total of 11 sessions. The interventions have been performed for the three types of stimulation by the same experimenters, in the same environment and during the same time period of inclusion.

The Institutional Review Board of Sud-Est V, Grenoble, France (No. 6705), approved the study on November 13, 2015. Written informed consent was obtained from all patients prior to enrolment. The study was registered in clinicaltrials.gov (NCT02817880).

### Study population

Patients with CRPS Type I were included if they met the following criteria: age between 18 and 80 years, disease duration for more than 1 year, a diagnosis confirmed by bone scintigraphy, pain intensity >3/10 at screening, no change in drug treatments during the last month and lack of response to conventional treatments. Patients were not included if they were pregnant or breastfeeding women, or if they had a CRPS diagnosis with presence of a neurological lesion, an intracranial ferromagnetic material or implanted device, a history of drug addiction, epilepsy, severe traumatic brain injury or neuropsychiatric comorbidities that could interfere with the assessment of outcomes.

### Randomization

After the baseline period, treatment allocation was made by the physician responsible for conducting the study at each centre using a secure Web-based random number generator (Research Electronic Data Capture system). Patients were randomly assigned to one of the three groups in a ratio of 1:1:1, with a random block size and stratified by two centres and affected limb (upper or lower limb).

### rTMS procedure

Stimulation was performed using a MagPro stimulator [MagVenture (distr. Mag2Health), Farum, Denmark] and either a flat B65 coil (MagVenture) in patients with upper limb pain or an angled B70 figure-of-eight coil (MagVenture) in patients with lower limb. Compared to flat figure-of-eight coils, the B70 coil is more powerful, leading to lower the resting motor threshold by 10–33%^22^ and stimulates deeper the motor cortex corresponding to lower limbs due to its angle of 150°.^23^

The motor cortical representation of the painful region was targeted using a TMS Navigator system, integrating individual brain MRI data (Localite, Sankt Augustin, Germany). Stimulation was performed at 10 Hz with an intensity set at 80% of the rest motor threshold (previously determined with motor evoked potential recording) and the coil held in posteroanterior orientation. Each rTMS session consisted of 40 trains of 5-s duration with inter-train interval of 25 s for a total of 2000 pulses in 20 min. This protocol is in line with the expert recommendations for safety.^24,25^

### tDCS procedure

Stimulation was performed using a Starstim neurostimulator (Neuroelectrics®, Barcelona, Spain) with saline-soaked surface sponge electrodes (35 cm²). The site of stimulation was determined according to the International 10–20 EEG System. The anode was placed over the motor cortex contralateral to pain, on C3/C4 in patients with upper limb pain and on C1/C2 in patients with lower limb. The cathode was placed over the supraorbital area ipsilateral to pain, on Fp1/Fp2. A constant current of 2 mA intensity was applied for 20 min.

### tsDCS procedure

Stimulation was performed using the Starstim neurostimulator (Neuroelectrics®, Barcelona, Spain) and the same parameters as for the tDCS protocol, except for the placement of the stimulation electrodes. These electrodes were placed longitudinally on the cervical or lumbar spine, according to a model study which demonstrated that this configuration had a relevant impact on the distribution of the induced electric field (EF) for the efficacy of tsDCS.^26^ In patients with upper limb pain, the anode was placed on the spinous processes of the C4–C5 vertebrae and the cathode along the column, spaced 8 cm below. In patients with lower limb pain, the anode was placed over the spinous processes of the L1–L2 vertebrae and the cathode along the column, spaced 8 cm below.

### Sample size calculation

Assuming a mean pain intensity measured on a 0–10 visual numerical scale (VNS) of 7 ± 1/10 in CRPS patients at baseline,^27,28^ a possible reduction of −2/10 at the end of the induction phase (Week 4) and of −1/10 at the end of the maintenance phase (Week 25) and a correlation of 0.7 or more between repeated measures, the expected sample size was 20 patients for each group, providing 80% power for a two-tailed test with significance level of 0.01 (adjusting for multiple comparisons) to demonstrate differences between pairs of groups (NQuery Advisor® 7.0). Finally, only 36 patients were enrolled before recruitment stopped after a 5-year inclusion period due to a recruitment challenge and the Covid-19 pandemia.

### Clinical outcomes/end-points

From the screening period (Week 4) to the end of follow-up (Week 25), patients filled a diary at home to measure their daily pain intensity on a VNS ranging from 0 (no pain) to 10 (the worst pain imaginable) and also to record the use of medication.

The primary end-point was the mean pain intensity during the week (weekly VNS pain score) measured over time (from Week 4 to Week 25).

Secondary end-points were the following clinical scores assessed on self-administered questionnaires completed by patients at Week 0 (pre-intervention visit), Week 13 (third month of treatment) and Week 25 (1-month post-treatment):

(1) the 12-item Short-Form Health Survey questionnaire (SF-12 questionnaire) to assess the health-related quality of life across two dimensions (Physical and Mental Components) with scores ranging from 0 to 100, a higher score indicating a better quality of life;^29^
(2) the hospital anxiety and depression scale (HADS) questionnaire to assess symptoms of anxiety and depression, with scores ranging from 0 to 21 for each subscale, a higher score indicating worse symptoms;^30^
(3) the Neuropathic Pain Symptom Inventory (NPSI) to assess the characteristics and impact of neuropathic pain, with total intensity score ranging from 0 to 100 and five sub-scores corresponding to various dimensions of pain [spontaneous superficial (burning) pain, deep (pressing) pain, paroxysmal pain, evoked pain and paraesthesia/dysaesthesia] ranging from 0 to 10, a higher score indicating greater symptom severity;^31^
(4) the Quick Disabilities of Arm, Shoulder and Hand (Q-DASH) scale to assess disability and symptoms affecting the upper limb, with a total intensity score ranging from 0 to 100, a higher score indicating more severe disability;^32^
(5) the Western Ontario and McMaster Universities Osteoarthritis Index for knee and hip (WOMAC) to assess disability and symptoms affecting the lower limb, with a total intensity score ranging from 0 to 100 (normalized for easy interpretation by multiplying total score by 100/96), a higher score indicating more severe disability.^33^

Of course, only patients with upper limb CRPS were required to complete the Q-DASH, and likewise only patients with lower limb CRPS were required to complete the WOMAC.

The overall effect of the stimulation was estimated by the patients according to the seven-point Clinical Global Impression—global Improvement (CGI-I) scale, from 1 (very much improved) to 7 (very much worsened) compared to the pre-treatment baseline period.^34^ Illness improvement rate was calculated as the percentage of patients improved. We also evaluated the responder rate at the end of follow-up (3-month post-treatment), according to a reduction of ≥30% from baseline regarding the mean intensity of pain on VNS.

Finally, at the last visit (post-treatment), the self-administered Comfort Rating Questionnaire (CRQ, first published by Palm *et al*.^35^) was completed to assess the various potential side-effects that occurred during or after the stimulation sessions (pain, tingling, burning, fatigue, nervousness, overall discomfort, sleep or concentration disturbances, alteration of visual or auditory perception and headache).

### Measurement of electrochemical skin conductance

Beyond the clinical impact of neuromodulation on pain and disability, we also assessed the possible modulation of the autonomic nervous system, which plays a key role in CRPS pathophysiology, by recording the sympathetic-driven ESC measured with the Sudoscan® device (Impeto Medical, Paris, France). Palmar and plantar ESC values were measured (in microSiemens, µS) at W0 (pre- treatment), W 13 (third month of treatment) and W25 (1-month post treatment). Sudoscan technology provides a quantitative assessment of small fibre neuropathy. During the test, patients were asked to stand for 2 min, with their palms and soles placed on large stainless steel electrode plates. A low direct current voltage (<4 V) was applied incrementally to the electrodes, generating a current proportional to the chloride ion flow extracted from the sweat glands innervated by small fibres. The ESC value was acquired for each foot and hand. The ESC was used to assess the skin conductance of affected limb, with higher measures indicating better conductance.^36^

### Statistical analysis

Continuous data are expressed as mean and standard deviation (SD) or median and interquartile range (IQR, 25–75th centiles), according to the normal or non-normal distribution, respectively, assessed by the Shapiro–Wilk test. Categorical data are expressed in numbers and percentages. One-way ANOVA, Kruskal–Wallis Test and Fisher’s exact test were used to compare outcomes at baseline across the three treatment groups.

Efficacy analyses were conducted on an intention-to-treat basis with missing data imputed as last-observation carried forward. All patients who received at least one session of treatment and completed at least one post-baseline weekly pain measurement were included in the intent-to-treat analysis. In addition, sensitivity analyses were conducted to explore the impact of missing values by performing a secondary analysis following the per-protocol principle that concerns only patients who completed each phase of study and had measurements for all pre-specified key times (Supplementary Fig. 1 and Supplementary Tables 3–6). We considered as key times the average of values collected in pain diary (weekly measurements) for baseline, Week 3 (end of the induction phase), Week 5 (after the end of first month of treatment), Week 13 (after the end of the third month of treatment), Week 21 (after the end of the treatment) and Week 25 (final follow-up, 1 month after the end of the treatment) (Fig. 1).

The primary end-point of the study (effects of treatments on pain) was examined by the use of two-way repeated measures ANOVA (rmANOVA) with treatment group (rTMS, tDCS and tsDCS) as the between-subject factor, time (week before treatment, Weeks 1–4 of induction phase, Weeks 5–20 of maintenance phase, Weeks 21–25 post-treatment) as the within-subject factor and the calculation of time-by-group interaction. Change from baseline over the time was also tested for each motor cortex neurostimulation group (rTMS and tDCS) compared with tsDCS group sequentially at a significance level of 0.017 (Bonferroni correction for three groups’ pairwise comparisons). The pairwise comparisons were tested only when the time-by-group interaction of the three groups over time was considered statistically significant. A significant time-by-group interaction would indicate that the change in pain intensity over time from baseline differed among groups. Sphericity was examined for all statistical analyses, and in case of non-sphericity, results were corrected according to the Greenhouse–Geisser method.

Within-group differences from the baseline (Week 1) to the follow-up periods (Weeks 3, 5, 13, 21 and 25) at each group were analysed using paired *t*-test. Between-group differences from the baseline period to each follow-up period were compared between the three groups using one-way ANOVA test. Bonferroni’s *post hoc* tests for comparisons to baseline were performed for significant main effects or interaction. Effect size was determined using Cohen’s d (Cohen’s criteria: small ≤0.2; moderate = 0.5; large ≥0.8) to compare the effects of the treatment group on the dependent variables. In addition, an analysis of covariance was implemented as a sensitivity analysis to compare groups at key time points, with baseline values used as covariates (Supplementary Tables 1 and 2). In order to simplify the report of the results, only the values related to pairwise comparisons tsDCS versus rTMS and tsDCS versus tDCS were reported.

The same analyses were used for pre-specified secondary end points (SF-12, HADS, NPSI, Q-DASH/WOMAC and ESC affected limb) with the factor time as a three-level variable (Fig. 1): baseline (visit 2), end of the third month of treatment (Visit 3) and 1-month post treatment (Visit 4). The Kruskal–Wallis test was used to compare the CGI-I and CRQ scale results across three treatment groups. For the responder rate, the proportion of participants in each group who demonstrated a reduction of ≥30% in pain from the baseline to the end of follow up was compared using the Fisher’s exact test.

The statistical significance level (α) for the comparison of three groups was set to 0.05, and the significance level (α) after Bonferroni’s correction for three groups was thus at 0.017. Analysis was conducted under blinded conditions using STATA 16.0 software (StatCorp, College Station, TX, USA).

## Results

### Flowchart of the study

**Figure 2:**
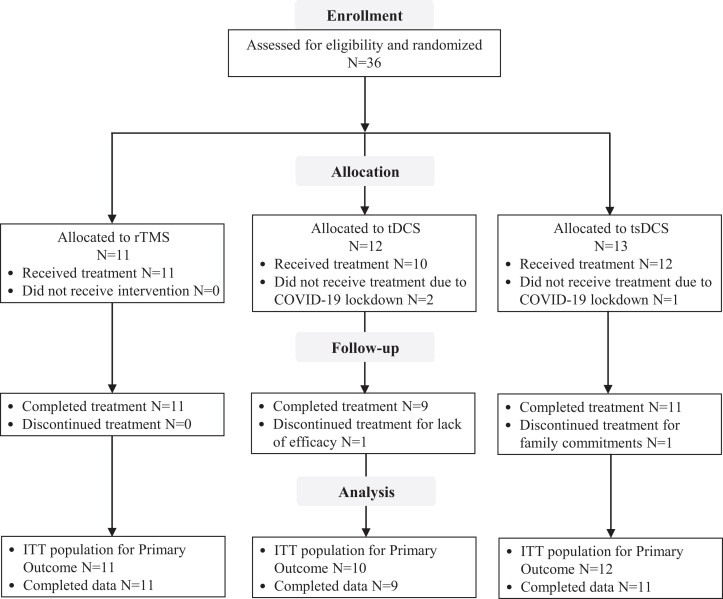
**Participant flow diagram.** Intent-to-treat (ITT) population included all randomized participants who received at least one treatment session and were assessed for baseline pain.

A total of 36 patients were enrolled and randomized to one of the three treatment groups (rTMS, *n* = 11; tDCS, *n* = 12; tsDCS, *n* = 13) (Fig. 2). Three patients (2 to tDCS group and 1 to tsDCS group) did not received treatment due to COVID-19 lockdown, and therefore, only 33 patients received treatment and were included in the intention-to-treat population. Overall, 94% (31/33, 11 to rTMS, 9 to tDCS and 11 to tsDCS) completed the entire follow-up assessment for the primary outcome. Two patients discontinued treatment, one in the tDCS group for lack of efficacy and one in the tsDCS group for family commitments.

### Baseline demographics and clinical characteristics

Baseline demographic and clinical characteristics were similar across treatment groups (*P* > 0.05) (Table 1). The mean age at enrolment was 46 years, 64% of patients were women and the median duration of pain syndrome was 24 months.

**Table 1:** Baseline characteristics of patients according to the allocated group

	Total (*N* = 33)	rTMS (*N* = 11)	tDCS (*N* = 10)	tsDCS (*N* = 12)
Age (years), mean (SD)	46.2 (12.2)	52.0 (9.6)	40.4 (12.5)	45.7 (12.6)
Female, *n* (%)	21 (64)	6 (54.5)	6 (60)	9 (75)
Disease history				
Pain syndrome duration (months), median (IQR)	24 (18–34)	24 (13–29)	22.5 (18–38)	24 (17.5–48)
Pain origin, *n* (%)				
Trauma (sprain, contusion, fracture)	14 (42.4)	6 (54.5)	2 (20.0)	6 (50.0)
Surgery	17 (51.5)	5 (45.5)	7 (70.0)	5 (41.7)
Other	2 (6.1)	0 (0.0)	1 (10.0)	1 (8.3)
Pain localization, lower limb, *n* (%)	21 (64)	5 (45.5)	7 (70)	5 (42)
Pain lateralization, left, *n* (%)	18 (54.5)	4 (36)	8 (80)	6 (50)
Drug treatment				
Current use of background drug therapy, *n* (%)	28 (85)	10 (91)	8 (80)	10 (83)
Number of background therapy used, median (IQR)	2 (2–3)	2 (2–3)	2.5 (1–3)	2.5 (2–3)
Type of background therapy used, *n* (%)				
Non-opioid analgesics	6 (18)	2 (18)	1 (10)	3 (25)
Weak opioid analgesics	16 (48.5)	7 (64)	5 (50)	4 (33)
Anti-epileptics	24 (73)	7 (64)	8 (80)	9 (75)
Antidepressants	20 (61)	7 (64)	5 (50)	8 (67)
Current use of pain crisis therapy, *n* (%)	29 (88)	9 (82)	9 (90)	11 (92)
Type of pain crisis therapy used, *n* (%)				
Non-opioid analgesics	19 (58)	5 (45)	6 (60)	8 (67)
Weak opioid analgesics	19 (58)	6 (54.5)	5 (50)	8 (67)
Strong opioid analgesics	3 (9)	0 (0)	2 (20)	1 (8)
Clinical assessment at baseline				
VNS pain, mean (SD)	6.1 (1.8)	5.4 (1.8)	6.3 (1.6)	6.6 (1.7)
SF-12 Physical component scale, mean (SD)	30.1 (5.4)	30.7 (4.8)	28.2 (6.0)	31.2 (5.4)
SF-12 Mental component scale, mean (SD)	38.1 (11.0)	40.9 (10.1)	39.9 (14.2)	34.0 (8.1)
HADS-anxiety, mean (SD)	11.3 (4.6)	10.0 (3.6)	11.5 (5.2)	12.3 (4.8)
HADS-depression, mean (SD)	9.5 (4.6)	8.5 (3.4)	9.3 (5.9)	10.8 (4.5)
NPSI total, mean (SD)	48.6 (22.0)	38.7 (16.6)	51.3 (26.0)	54.6 (21.4)
Q-DASH/WOMAC, mean (SD)	65.2 (14.9)	63.1 (15.8)	62.3 (14.8)	69.6 (14.4)
ESC affected limb (μS), mean (SD)	72.3 (16.8)	74.9 (15.3)	71.2 (19.1)	70.5 (17.6)

SNRI, serotonin-norepinephrine reuptake inhibitor; SSRI, selective serotonin reuptake inhibitors; NSAIDs, non-steroidal anti-inflammatory drugs; VNS, Visual Numeric Scale (0–10); SF-12, 12-item Short-Form Health Survey questionnaire (0–100): Physical and Mental subscales; HADS, hospital anxiety and depression scale (0–21): Anxiety and Depression subscales; NPSI, Neuropathic Pain Symptom Inventory (total score 0–100, sub-scores 0–10); Q-DASH, Quick Disabilities of Arm, Shoulder and Hand scale normalized to 100 (score range 0–100); WOMAC, Western Ontario and McMaster Universities Osteoarthritis Index for knee and hip (normalized 0–100); ESC, electrochemical sweat conductance obtained with SUDOSCAN®. Data are expressed as mean (SD) or median (IQR), according to the normal or non-normal distribution, assessed by the Shapiro–Wilk test. Statistics: one-way ANOVA or Kruskal–Wallis test was used for continuous data and Fisher’s exact test for categorical variables. All *P*-values are >0.05.

### Primary outcome: change in weekly VNS pain score

The primary outcome was the change in weekly VNS pain score over time from baseline (pre-treatment, Week 0) to 1-month post treatment (Week 25).

**Figure 3:**
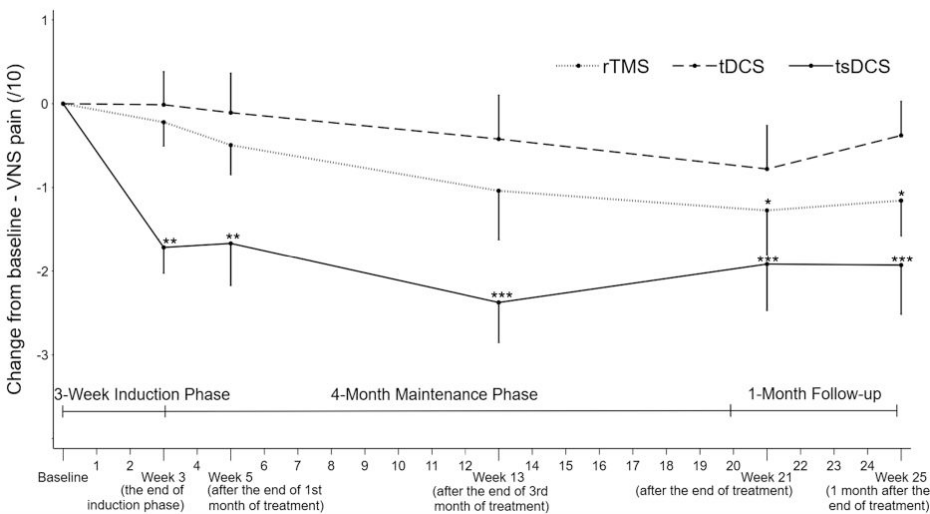
**Mean change over time in the visual numerical pain score according to the type of stimulation.** The line graph represents the mean values with standard errors bars in the intention-to-treat population. Changes from baseline in mean pain intensity rated on a 0–10 VNS are plotted over the 25 weeks of follow-up for the three groups of patients with complex regional pain syndrome treated by high-frequency rTMS, tDCS or tsDCS. The two-way rmANOVA revealed significant time-by-group interaction (*P* = 0.014, estimated by the use of rmANOVA model including stimulation group, time and the interaction of stimulation group with time), with Bonferroni’s *post hoc* tests compared to baseline showing VNS pain score decrease in the tsDCS group at all time points and in the rTMS group at the last two time points (*P < 0.05, **P < 0.01, ***P < 10^−3^).

In Fig. 3, the mean change from baseline is plotted against time for each treatment group at the various time points of assessment: Week 3 (at the end of induction phase), Week 5 (after 1 month of treatment), Week 13 (after 3 months of treatment), Week 21 (at the end of maintenance therapy) and Week 25 (final follow-up, 1 month after the end of treatment). The two-way rmANOVA analysis revealed significant time effect for rTMS group [F_(25,250)_ = 2.39, *P* = 0.0004] and tsDCS group [F_(25,275)_ = 2.65, *P* = 0.0001] but not for tDCS group [F_(25,225)_ = 0.77, *P* = 0.779]. *Post hoc* tests of rmANOVA showed a significant reduction in the weekly VNS pain score at all time points compared to baseline in the tsDCS group (Week 3: *P* = 0.001; Week 5: *P* = 0.002; Week 13: *P* < 10^−3^; Week 21: *P* < 10^−3^; Week 25: *P* = < 10^−3^), at the last two time points in the rTMS group (Week 21: *P* = 0.010; Week 25: *P* = 0.029), but at no time point in the tDCS group (Fig. 3).

The two-way rmANOVA analysis revealed significant time-by-group interaction in the three-group analysis [F_(50,750)_ = 1.52, *P* = 0.014] and the two-group analyses regarding tsDCS versus rTMS [F_(25,525)_ = 1.74, *P* = 0.015] and tsDCS versus tDCS [F_(25, 525)_ = 1.73, *P* = 0.016]. *Post hoc* pairwise comparisons showed a significant decrease from baseline of the VNS pain score at Week 3 (end of induction phase) regarding tsDCS versus rTMS (−1.7 ± 1.1 versus −0.2 ± 0.9, effect size = −1.5, *P* = 0.008) and tsDCS versus tDCS (−1.7 ± 1.1 versus 0.0 ± 1.2, effect size = −1.5, *P* = 0.003) (Table 2). A tendency towards a better efficacy of tsDCS versus tDCS was also observed at Week 5 (−1.7 ± 1.8 versus −0.1 ± 1.5, effect size = −1.0, *P* = 0.063) and Week 13 (−2.4 ± 1.7 versus −0.4 ± 1.7, effect size = −1.2, *P* = 0.043) (Table 2).

**Table 2:** Primary outcome: visual numeric pain scale

	tsDCS	rTMS	tDCS	tsDCS versus rTMS	tsDCS versus tDCS
BL	6.6 (1.7)	5.4 (1.8)	6.3 (1.6)		
W3 to BL	−1.7 (1.1), d = −1.6	−0.2 (0.9), d = −0.2	0.0 (1.2), d = 0.0	−1.5 (−2.6 to −0.3), d = −1.5, *P* = 0.008	−1.7 (−2.9 to −0.5), d = −1.5, *P* = 0.003
W5 to BL	−1.7 (1.8), d = −1.0	−0.5 (1.2), d = −0.4	−0.1 (1.5), d = −0.1	−1.2 (−2.8 to 0.4), d = −0.8, *P* = 0.210	−1.6 (−3.2 to 0.1), d = −1.0, *P* = 0.063
W13 to BL	−2.4 (1.7), d = −1.5	−1.0 (1.9), d = −0.5	−0.4 (1.7), d = −0.6	−1.3 (−3.2 to 0.5), d = −0.7, *P* = 0.234	−2.0 (−3.9 to −0.1), d = −1.2, *P* = 0.043
W21 to BL	−1.9 (1.9), d = −1.0	−1.2 (1.4), d = −0.7	−0.4 (1.3), d = −0.9	−0.6 (−2.5 to 1.2), d = −0.4, *P* = 0.999	−1.1 (−3.1 to 0.8), d = −0.6, *P* = 0.440
W25 to BL	−1.9 (2.0), d = −1.0	−1.2 (1.4), d = −0.8	−0.4 (1.3), d = −0.9	−0.8 (−2.5 to 0.9), d = −0.4, *P* = 0.796	−1.6 (−3.3 to 0.2), d = −0.9, *P* = 0.102

BL, baseline; W, week; *d*, Cohen’s d, effect size. Data are expressed as mean ± SD or mean with 95% CI. A negative change within groups means improvement. Between-group differences were calculated as tsDCS group results: a negative difference favours the tsDCS group. In the right columns, pairwise comparisons were analysed by Bonferroni *post hoc* tests.

In addition, the differences between groups when the means were adjusted from baseline values were in favour of tsDCS at Week 3 (difference versus rTMS: −1.4; 95% CI: −2.7 to −0.2; *P* = 0.019 and difference versus tDCS: −1.7; 95% CI: −2.9 to −0.5; *P* = 0.004), at Week 5 (difference versus tDCS: −1.5; 95% CI: −3.1 to 0.1; *P* = 0.077) and at Week 13 (difference versus tDCS: −1.7; 95% CI: −3.6 to 0.2; *P* = 0.098).

When each treatment effect was compared numerically using Cohen’s d, the post treatment effect was the largest in the tsDCS group up to Week 13 (d values in the tsDCS, rTMS, tDCS groups, respectively at Week 3: −1.6, −0.2, 0.0; Week 5: −1.0, −0.4, −0.1; Week 13: −1.5, −0.5, −0.6; Week 21: −1.0, −0.8, −0.9; Week 25: −1.0, −0.8, −0.9).

Similarly, the proportion of patients with at least 30% reduction in weekly VNS pain score from baseline (responders) was significantly higher in the tsDCS group (73%) compared to the rTMS (18%) and tDCS (11%) groups at Week 13 (*P* = 0.010). On the other hand, this proportion was significantly higher in the rTMS group (54%) than in the tsDCS (27%) and tDCS (0%) groups at the end of follow-up (Week 25) (*P* = 0.035).

### Secondary outcomes: self-administered clinical questionnaires

For the SF-12 Mental Component score, the two-way rmANOVA analysis revealed significant time effect only for the tsDCS group [F_(2,22)_ = 6.05, *P* = 0.012] and not for rTMS group [F_(2,20)_ = 0.41, *P* = 0.596] or tDCS group [F_(2,18)_ = 1.87, *P* = 0.195]. *Post hoc* tests of rmANOVA showed a significant improvement only at Week 25 compared to baseline in the tsDCS group (Week 13: *P* = 0.060; Week 25: *P* = 0.017) but at no time points in the other groups.

For the SF-12 Physical Component score, the two-way rmANOVA analysis revealed no significant time effect.

For the HADS-anxiety score, the two-way rmANOVA analysis revealed significant time effect for the tsDCS group [F_(2,22)_ = 5.32, *P* = 0.013] and tDCS group [F_(2,18)_ = 7.06, *P* = 0.010] but not for rTMS group [F_(2,20)_ = 2.64, *P* = 0.100]. *Post hoc* tests of rmANOVA showed a significant reduction only at Week 25 compared to baseline in the tsDCS group (*P* = 0.007) and in the tDCS group (*P* = 0.006) but at no time points in the rTMS group.

For the HADS-depression score, the two-way rmANOVA analysis revealed significant time effect only for the tsDCS group [F_(2,22)_ = 9.38, *P* = 0.003] and not for rTMS group [F_(2,20)_ = 2.13, *P* = 0.776] or tDCS group [F_(2,18)_ = 1.38, *P* = 0.276]. *Post hoc* tests of rmANOVA showed a significant improvement at all-time points compared to baseline in the tsDCS group (Week 13: *P* = 0.007; Week 25: *P* = 0.002) but at no time points in the other groups.

For the NPSI score, the two-way rmANOVA analysis revealed significant time effect for the tsDCS group [F_(2,22)_ = 8.19, *P* = 0.002] and rTMS group [F_(2,20)_ = 7.71, *P* = 0.003] but not for tDCS group [F_(2,18)_ = 2.86, *P* = 0.083]. *Post hoc* tests of rmANOVA showed a significant reduction only at Week 25 compared to baseline in the tsDCS group (*P* = 0.002) and in the rTMS group (*P* = 0.003) but at no time points in the tDCS group.

For the Q-DASH/WOMAC score, the two-way rmANOVA analysis revealed significant time effect only for the tsDCS group [F_(2,22)_ = 3.68, *P* = 0.042] and not for rTMS group [F_(2,20)_ = 0.73, *P* = 0.496] or tDCS group [F_(2,18)_ = 0.34, *P* = 0.715]. *Post hoc* tests of rmANOVA showed a significant improvement only at Week 25 compared to baseline in the tsDCS group (*P* = 0.050) but at no time points in the other groups.

The two-way rmANOVA revealed no significant time-by-group interaction in the three-group analysis regarding any of the clinical questionnaires used in this study (SF-12, HADS, NPSI, Q-DASH/WOMAC), although a tendency towards a significant interaction was observed for SF-12 Mental Component score [F_(4,60)_ = 2.24, *P* = 0.083] (Table 3). Moreover, *post hoc* pairwise comparisons showed a significant improvement of this score regarding tsDCS versus rTMS at Week 13 (6.8 ± 9.0 versus −2.0 ± 4.9, effect size = 1.2, *P* = 0.019) and a tendency at Week 25 (8.3 ± 9.8 versus −1.2 ± 9.3, effect size = 1.0, *P* = 0.081) (Table 3). For the other clinical variables, *post hoc* pairwise comparisons only showed at Week 13 a tendency towards a better efficacy of tsDCS versus rTMS regarding HADS-anxiety score (−2.0 ± 3.0 versus 0.2 ± 1.6, effect size = −0.9, *P* = 0.095) and versus tDCS regarding HADS-depression score (−2.4 ± 2.9 versus 0.0 ± 2.1, effect size = −1.0, *P* = 0.072) (Table 3).

**Table 3:** Secondary outcomes: clinical questionnaires

	tsDCS	rTMS	tDCS	tsDCS versus rTMS	tsDCS versus tDCS
**SF-12 Mental Component** (F_time × stimulation 4,60_ = 2.24; *P* = 0.083)					
BL	33.9 (8.0)	40.9 (10.0)	39.7 (14.2)		
W13 to BL^a^	6.8 (9.0), d = 0.8	−2.0 (4.9), d = −0.4	1.6 (6.6), d = 0.2	8.8 (1.2 to 16.3), d = 1.2, *P* = 0.019	5.2 (−2.6 to 12.9), d = 0.6, *P* = 0.305
W25 to BL^a^	8.3 (9.8)*, d = 0.8	−1.2 (9.3), d = −0.1	5.7 (10.1), d = 0.6	9.4 (−0.8 to 19.7), d = 1.0, *P* = 0.081	2.6 (−8.0 to 13.1), d = 0.3, *P* = 0.999
**SF-12 Physical Component** (F_time × stimulation 4,60_ = 0.50; *P* = 0.697)					
BL	31.2 (5.202)	30.6 (4.9)	28.3 (6.0)		
W13 to BL	0.5 (4.2), d = 0.1	1.5 (3.6), d = 0.4	−0.2 (3.4), d = −0.1	−1.0 (−4.5 to 2.4), d = −0.3, *P* = 0.530	0.7 (−2.8 to 4.2), d = 0.2, *P* = 0.678
W25 to BL	2.5 (5.3), d = 0.5	3.5 (7.8), d = 0.5	0.4 (3.2), d = 0.1	−1.0 (−6.7 to 4.7), d = −0.2, *P* = 0.709	2.1 (−1.9 to 6.1), d = 0.5, *P* = 0.289
**HADS-anxiety** (F_time × stimulation 4,60_ = 1.68; *P* = 0.175)					
BL	12.3 (4.8)	10.0 (3.6)	11.5 (5.2)		
W13 to BL^a^	−2.0 (3.0), d = −0.7	0.2 (1.6), d = 0.1	−2.0 (2.0), d = −1.0	−2.2 (−4.6 to 0.3), d = −0.9, *P* = 0.095	0.0 (−2.5 to 2.5), d = 0.0, *P* = 0.999
W25 to BL	−3.4 (4.1)**, d = −0.8	−1.0 (2.0), d = −0.5	−2.8 (3.0)**, d = −0.9	−2.4 (−5.2 to 0.4), d = −0.8, *P* = 0.089	−0.6 (−3.8 to 2.9), d = −0.2, *P* = 0.694
**HADS-depression** (F_time × stimulation 4,60_ = 2.03; *P* = 0.109)					
BL	10.7 (4.5)	8.4 (3.4)	9.3 (5.9)		
W13 to BL^a^	−2.4 (2.9)**, d = −0.8	−0.3 (2.0), d = 0.1	0.0 (2.1), d = 0.0	−2.1 (−4.7 to 0.4), d = −0.9, *P* = 0.116	−2.4 (−5.0 to 0.2), d = −1.0, *P* = 0.072
W25 to BL	−2.8 (2.5)**, d = −1.1	−0.45 (3.9), d = −0.1	−1.0 (1.9), d = −0.5	−2.4 (−5.2 to 0.4), d = −0.7, *P* = 0.095	−1.8 (−3.9 to 0.2), d = −0.8, *P* = 0.075
**NPSI total** (F_time × stimulation 4,60_ = 0.69; *P* = 0.599)					
BL	54.6 (21.4)	38.8 (15.8)	51.3 (26.0)		
W13 to BL	−12.0 (20.0), d = −0.6	−6.4 (10.2), d = −0.6	−6.8 (13.5), d = −0.5	−5.6 (−19.6 to 8.3), d = −0.4, *P* = 0.410	−5.2 (−20.7 to 10.3), d = −0.3, *P* = 0.492
W25 to BL	−20.0 (16.6)**, d = −1.2	−12.8 (11.8)**, d = −1.1	−10.6 (14.4), d = −0.7	−7.2 (−19.8 to 5.4), d = −0.5, *P* = 0.249	−9.4 (−23.4 to 4.6), d = −0.6, *P* = 0.176
**Q-DASH/WOMAC** (F_time × stimulation 4,60_ = 0.72; *P* = 0.580)					
BL	69.6 (14.4)	63.1 (15.8)	62.4 (14.8)		
W13 to BL	−8.2 (15.7), d = −0.5	−2.3 (12.6), d = −0.2	−0.1 (13.0), d = 0.0	−5.9 (−18.3 to 6.5), d = −0.4, *P* = 0.334	−8.1 (−21.1 to 4.9), d = −0.6, *P* = 0.207
W25 to BL	−10.0 (14.0), d = −0.7	−4.7 (16.2), d = −0.3	−2.7 (12.9), d = −0.2	−5.3 (−18.4 to 7.8), d = −0.4, *P* = 0.409	−7.3 (−19.4 to 4.8), d = −0.5, *P* = 0.224

BL, Baseline; W, week; d, Cohen’s d, effect size; SF-12, 12-item Short-Form Health Survey questionnaire (0–100): Physical and Mental subscales; HADS, hospital anxiety and depression scale (0–21): Anxiety and Depression subscales; Q-DASH, Quick Disabilities of Arm, Shoulder and Hand scale (0–100); WOMAC, Western Ontario and McMaster Universities Osteoarthritis Index for knee and hip (0–100). In the right columns, pairwise comparisons were analysed by Bonferroni *post hoc* tests. Data are expressed as absolute change from baseline (mean ± SD). Bonferroni’s *post hoc* test compared to baseline: *P < 0.05; **P < 0.01; ***P < 0.001.

a Pairwise comparisons were analysed by Bonferroni’s post hoc tests (95% CI and *P*) if *P* of difference between the three groups were ≤0.100.

### Secondary outcomes: impact on Clinical Global Impression—global Improvement scale

**Figure 4:**
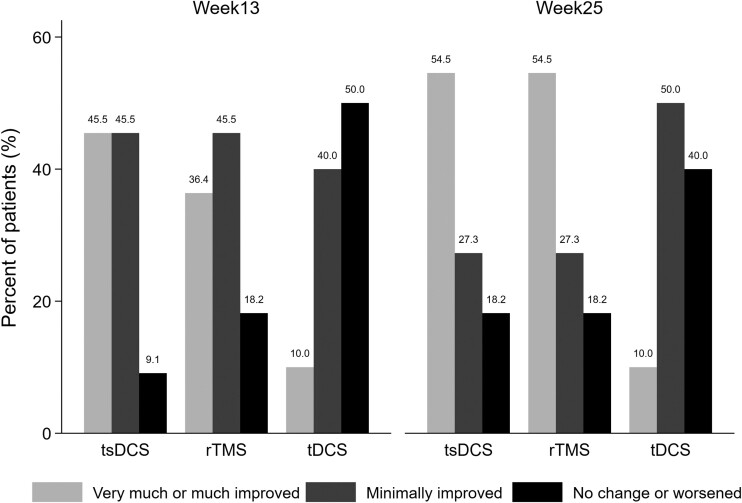
**Response rate on the clinical global impression improvement scale (CGI-I).** The bars represent the percentages of patients very much or much improved (CGI-I 1–2), minimally improved (CGI-I 3) and without change or worsened (CGI-I ≥ 4). Percentages are shown above the bars.

On the CGI-I scale, a significant difference between groups was observed in the improvement rate at Week 13 (*P* = 0.049) but not at Week 25 (*P* = 0.139). The rate of patients with at least minimal improvement was higher in the tsDCS versus tDCS group at Week 13 (*P* = 0.024), but not at Week 25 (*P* = 0.124) (Fig. 4). No difference was observed between other treatment groups.

### Secondary outcomes: impact on side-effects (Comfort Rating Questionnaire)

No significant difference in CRQ sum scores or any specific CRQ item was found between treatment groups (data not shown). No adverse effects were reported during or following any of the three interventions.

### Secondary outcomes: ESC measurements

On the ESC at the limb affected, no significant change was observed over time at Week 13 or Week 25 in any treatment groups, and no difference between the groups was neither observed (data not shown). However, at baseline, ESC was reduced at the limb affected by CRPS compared to the non-affected limb [mean (µS) ± SD: 72.3 ± 16.8 versus 75.0 ± 15.5, *P* = 0.015]. In addition, the patients with at least 30% reduction in weekly VNS pain score at Day 90/Week 13 from baseline (responders) had lower ESC values at the affected limb compared to the other patients (non-responders) at baseline [mean (µS) ± SD: 61.6 ± 19.0 versus 78.2 ± 12.5, *P* = 0.007] and also a significant ESC increase at Day 90/Week 13 (78.4 ± 18.4 and 67.4 ± 14.1, *P* = 0.007).

## Discussion

Overall, this study shows a better efficacy of spinal stimulation by means of tsDCS compared to motor cortex stimulation techniques (rTMS, tDCS) to produce an analgesic effect in patients with chronic refractory CRPS. This improved efficacy was also observed more rapidly, during the induction phase. In the longer term, rTMS also showed significant efficacy, but not tDCS. This is one of the first studies to show the efficacy of tsDCS in a chronic pain syndrome, which is also a recognized indication for implanted SCS.^15^

Only a few studies have been reported to date on the use of non-invasive neuromodulation in patients with CRPS, as recently reviewed.^37^ Regarding rTMS, beyond a proof-of-principle trial based on a single session,^38^ only two studies showed some analgesic effects of 10Hz-rTMS applied to the motor cortex in patients with CRPS.^28,39^ First, in a sham-controlled study, Picarelli *et al*.^28^ applied rTMS as an add-on intervention to standard pharmacological and rehabilitation therapy for 10 consecutive sessions in 23 patients with CRPS Type I (12 active, 11 sham). Not only pain intensity but also functional and affective scores were reduced during the rTMS protocol, but the effects vanished soon (<1 week) after the stimulation period. However, there was a large inter-individual variation in the response duration, and one patient was completely relieved up to 3 months after rTMS. Second, in an open-label study, Gaertner *et al*.^39^ investigated the effects of five daily rTMS sessions primed by a sequence of intermittent theta burst stimulation in 12 patients with CRPS of Type I or II. Pain intensity score was decreased by more than 30% in 58% of the patients (7/12) at the end of the stimulation protocol. Among these seven patients, three remained improved for 3–4 weeks and two for 3–4 months beyond the rTMS protocol.

Regarding tDCS, beyond two single case reports,^40,41^ only one randomized sham-controlled study was reported.^42^ In a series of 22 patients with CRPS Type I (11 active, 11 sham), Lagueux *et al*.^42^ applied anodal tDCS over the motor cortex for five consecutive days during the first 2 weeks of a therapy based on a technique of graded motor imagery and once a week during 4 subsequent weeks of graded motor imagery therapy. No significantly greater pain reduction was observed in the group of patients receiving active tDCS versus sham tDCS, while some differences were observed between the two groups in terms of pain catastrophizing and anxiety, but not at a clinically meaningful level.^42^ However, the effect of a protocol of tDCS performed alone has never been assessed in the context of CRPS.

Regarding non-invasive stimulation performed on non-brain structures in patients with CRPS, there are various published studies using repetitive peripheral magnetic stimulation or transcutaneous electrical nerve stimulation (TENS) on neuromuscular structures (reviewed in^37^), but not on spinal structures. In particular, the analgesic effect of tsDCS has never been evaluated in patients with CRPS. Anodal tsDCS has mainly been applied to modulate experimentally induced pain in healthy subjects. For example, anodal tsDCS has been shown to modulate pain ratings or thresholds in response to pressure,^43^ heavy mechanical pinprick^44^ or other types of nociceptive stimuli,^45^ but without affecting hyperalgesia induced by electrical high-frequency stimulation.^46^ Neurophysiological correlates of these analgesic effects were also investigated, either at the segmental level of spinal nociceptive integration by assessing changes induced by tsDCS in the nociceptive withdrawal flexion reflex (NWFR)^47,48^ or along the spinothalamic nociceptive pathways by recording laser-evoked cortical potentials.^19,45,49^

On the other hand, as mentioned in the introduction, we found only two clinical studies reporting the analgesic effect of repeated sessions of tsDCS in patients with chronic pain.^20,21^ First, Guidetti *et al*.^21^ applied anodal tsDCS (2.5 mA) in 16 patients with chronic pain of different aetiology for five daily sessions within a week. The anode was placed over the tenth thoracic vertebra and the cathode over the somatosensory cortex. The pain condition was related to lumbosacral radiculopathy in nine patients, herpes zoster in two patients, diabetes in two patients or multiple sclerosis in one patient. The design of the study was a randomized, sham-controlled crossover trial. Compared to sham, anodal tsDCS decreased pain intensity and NPSI scores at 1 month after the week of stimulation, in correlation with a reduction of NWFR area. Second, Berra *et al*.^20^ applied anodal tsDCS (2 mA) in 33 patients with chronic neuropathic pain related to multiple sclerosis for 10 daily sessions within 2 weeks. The anode was placed over the tenth thoracic vertebra and the cathode over the right shoulder. The design of the study was a randomized, sham-controlled parallel-arm trial (19 active, 14 sham). Compared to sham, anodal tsDCS decreased NPSI score from the end of the stimulation period up to 1 month after, while the NWFR tended to be also reduced.

Our results are consistent with these precedents, but extend the findings over the long term according to a 5-month stimulation protocol based on 23 tsDCS sessions. This point is of importance, since most rTMS and tDCS studies are of short duration, in particular the very few studies that compared the analgesic effects of rTMS versus tDCS in patients.^50,51^ The rare studies reporting long-term analgesic efficacy of rTMS^52-59^ and tDCS^60,61^ have been not performed in the context of CRPS.

On the other hand, there was a striking methodological difference in our study compared to previous ones, which was the montage including the placement of both anode and cathode along the spine. Such a ‘spinal montage’ is most relevant for focusing the induced EF in the spinal cord as recently modelled.^62^ Indeed, the maximum EF magnitude resulting from this montage is predicted to lie in the spinal segment comprised between the anode and the cathode in human models.

Even though the dorsal horns are probably the site most strongly modulated by tsDCS, the diffusion of EF in the transverse (horizontal) plane and the nature of the neuronal structures affected remain speculative. Modelling studies have, however, improved the understanding of the spatial distribution of the current density generated by tsDCS.^63,64^ There is further evidence that tsDCS can induce persistent changes in the trans-synaptic properties of spinal neurons.^64^ In the context of nociceptive information processing, the neuromodulatory effect of tsDCS is primarily segmental and most likely associated with changes in the synaptic efficiency of input pathways at the level of the dorsal horn.^49^ Also, long-term synaptic changes (depression or potentiation) can occur in the spinal cord in a manner dependent on the polarity of stimulation, anodal or cathodal.^65^ Like tDCS,^66^ tsDCS is likely to modify glutamatergic neurotransmission, notably involving N-methyl-D-aspartate receptors, which may have an important role in prolonging segmental changes in spinal neuronal activity in the long term. For example, it was found that the perception of provoked pain was already reduced during the application of tsDCS but became more pronounced during the 30 min following its discontinuation, although inter-individual variation was large.^47^

However, the mechanisms of action might not be strictly segmental and spinal, as tsDCS can also modulate various neural structures and neurotransmitter systems through ascending spinal pathways to the brain, including brainstem or thalamo-cortical networks. Indeed, there is evidence that the changes in excitability properties of neurons induced by tsDCS extent to corticospinal tracts or even intracortical circuits, as revealed by TMS excitability studies^67-69^ or fMRI of brain connectivity.^70^ In particular, fMRI showed that anodal tsDCS resulted in a decreased connectivity between the somatosensory cortex and the posterior insula and between the thalamus and the anterior cingulate cortex, while cortico-thalamic connectivity was increased.^70^ Regarding TMS variables, tsDCS was found to modulate resting motor cortex threshold,^68^ short-interval intracortical inhibition (SICI)^68^ and interhemispheric functional connection (transcallosal conduction time, TCT)^69^ in a polarity-specific manner: resting motor threshold, SICI and TCT are increased by anodal tsDCS, while cathodal tsDCS produces rather opposite effects.

On the other hand, CRPS is characterized by various alterations in TMS parameters of cortico-spinal excitability (reviewed in^71^), especially a reduced SICI. These literature data therefore offer a pathophysiological rationale for the efficacy of anodal tsDCS, which would be to restore a deficient SICI in patients with chronic pain related to CRPS. Restoration of SICI has been the subject of several publications regarding the analgesic efficacy of neuromodulation techniques, with a proven correlation between SICI improvement and pain relief following motor cortex rTMS,^72^ anodal tDCS^73^ or even repetitive peripheral magnetic stimulation.^74^ This hypothesis merits specific investigation in future work on the analgesic role of tsDCS in patients with chronic pain.

Another interesting end-point is the measurement of ESC, the reduction of which for the affected limb at baseline seeming to be predictive of good pain relief under treatment. Moreover, successful neuromodulation therapy was associated with a significant increase (normalization) of initially reduced ESC. This result illustrates the involvement of the sympathetic nervous system in CRPS and its ‘cross-talk’ with somatosensory processing.^75^ At least a subset of patients had evidence of sympathetic deficit (reduced ESC) in the affected limb and cortical or spinal neuromodulation may have corrected this central sympathetic dysfunction in association with pain relief. However, this finding was not specific to the effect of tsDCS.

Other mechanisms of action may be involved in the analgesic effect of tsDCS. Depending on the orientation and intensity of the induced EFs, a steady direct current delivered by repeated daily sessions of tDCS over 20-min duration may guide and stimulate the migration or proliferation of inflammatory or glial cells or the outgrowth of neurites by specific actions on various cytokines or neurotrophins.^10,76^ Through the application of tsDCS, segmental modulation of inflammatory response and healing or regeneration of neural tissue may have beneficial impact on the activity and plasticity of spinal neurons. Therefore, using repeated sessions of tsDCS, pain reduction and clinical improvement may be related to various molecular or cellular interactions beyond involving a segmental or bottom-up modulation of synaptic plasticity and neural network activities, as suggested by a recent experimental study in a rat model of chronic pain.^77^ All of these changes can contribute to a progressive positive outcome in the long term beyond the stimulation period, as also illustrated by the impact of tsDCS procedure in the rehabilitation of motor control disorders, for example.^78^

As mentioned above, the potential benefit of tsDCS in CRPS echoes the fact that CRPS is a classic indication for invasive SCS.^2,15,79^ The mechanisms of action of SCS are multiple,^80^ probably as for tsDCS, and SCS parameters of stimulation are currently diversifying, with new modes, including broadly covering or highly focused high-frequency, burst or paraesthesia-free stimulation.^81^ Like conventional SCS, these new modes may also be effective in treating CRPS.^82^ On the other hand, implanted dorsal root ganglion stimulation (DRGS) has recently been shown to work even better and longer than implanted SCS to improve patients with CRPS.^83,84^ It is unclear from modelling studies whether tsDCS can act on dorsal roots as it does on dorsal horns. However, at least for the lower limbs, with the anode at the level of the L1–L2 vertebrae and the cathode 8 cm below, our tsDCS montage is more suitable for stimulating the cauda equina, but also the dorsal root ganglia, than the lumbar spinal cord corresponding to the painful territory of the lower limb. Whatever the exact target, our study shows that a non-invasive approach to spinal stimulation could be an alternative solution to invasive SCS for the treatment of chronic pain. This is already the case for rTMS which has replaced epidural stimulation of the motor cortex in clinical practice for the treatment of neuropathic pain.^6^ However, while rTMS and epidural MCS most likely share the same mechanisms of action for producing pain relief,^85^ it is certain that the pulsed electrical stimuli delivered by implanted SCS or DRGS have a markedly different impact at the neuronal level compared to the constant current delivered by tsDCS. This is notably due to the fact that SCS or DRGS generate action potentials and not tsDCS.^10^

As a non-invasive neuromodulation technique, the advantage of tsDCS, like TENS, is that it can be performed at home, without any requirement for surgically implantable devices, unlike SCS or DRGS. An intermediate solution could be the use of percutaneous direct current stimulation, currently under development.^86^

This pioneering study has various limitations, especially the absence of sham control, the relatively small sample size and the fact that the CRPS severity score,^87,88^ which is capable of defining a clinically relevant change in CRPS symptomatology, was not used, as well as objective neurophysiological methods to assess sensory and autonomic symptoms.

Regarding tDCS, this study showed the inefficacy of tDCS with a conventional bipolar montage in relieving chronic pain associated with CRPS. However, the conventional bipolar montage used here is not at all focal and probably led to various changes in axonal excitability in a large region of the cortex and not strictly limited to the region of M1 corresponding to the painful limb, unlike rTMS using a focal coil and neuronavigated targeting. Also, differences in spatial interactions with the cerebral neural networks related to the geometry of the induced current, even beyond the differences in mechanisms of action inherent in these neuromodulation techniques, could explain the difference in efficacy between the two cortical stimulation methods used in this study. Thus, these results do not rule out a potential value of tDCS with other montages (high definition or multisite) or on other cortical (e.g. somatosensory or dorsolateral prefrontal cortex^89,90^) or cerebellar^91^ targets.

Anyway, these results pave the way for the use of cervical or lumbar anodal tsDCS as a therapeutic technique for providing pain relief in patients with CRPS. This is especially possible to consider given the improvement obtained in the absence of side-effects and the ease of performing the technique. A future controlled study is awaited to confirm the drastic effect observed here compared to placebo stimulation. Other evaluation methods, both clinical and neurophysiological ones, should also be added to better determine the mechanisms of action of tsDCS. These mechanisms could be segmental or supraspinal, related to modulations of sensory, nociceptive or sympathetic neuronal circuits, to neuroplasticity or to molecular or cellular neurotrophic effects associated with longer-term improvement.

## Supplementary Material

fcad191_Supplementary_Data

## Data Availability

Raw data that support the findings of this study are available from the corresponding author, upon reasonable request.
