# Supplementary material for: Long-term analgesic effect of trans-spinal direct current stimulation compared to non-invasive motor cortex stimulation in complex regional pain syndrome
Source: Brain Commun. 2023 Jul 1;5(4):fcad191. doi: 10.1093/braincomms/fcad191 (PMC10400160; doi:10.1093/braincomms/fcad191)
Supplement: fcad191_Supplementary_Data [file fcad191_supplementary_data.docx]

**Supplementary Appendix**

This appendix has been provided by the authors to give readers additional information about their work.

Supplement to: Hodaj H, et al. “Long-term analgesic effect of trans-spinal direct current stimulation compared to non-invasive motor cortex stimulation in complex regional pain syndrome.”

**Table of contents**

[**Sensitivity Analysis** 3](#_Toc115084079)

[**Supplementary Figure 1** 3](#_Toc115084080)

[**Supplementary Table 1** 4](#_Toc115084081)

[**Supplementary Table 2** 5](#_Toc115084082)

[**Supplementary Table 3** 6](#_Toc115084083)

[**Supplementary Table 4** 7](#_Toc115084084)

[**Supplementary Table 5** 9](#_Toc115084085)

[**Supplementary Table 6** 10](#_Toc115084086)

# **Sensitivity Analysis**

Sensitivity analyses for the primary endpoint and continuous secondary endpoints include the same analysis using the per-protocol population.

In addition, an analysis of covariance (ANCOVA) was implemented as a sensitivity analysis to compare groups at key time points, with baseline values used as covariates.

# **Supplementary Figure 1. Mean change over time in the visual numeric pain score according to the type of stimulation in per-protocol population.**


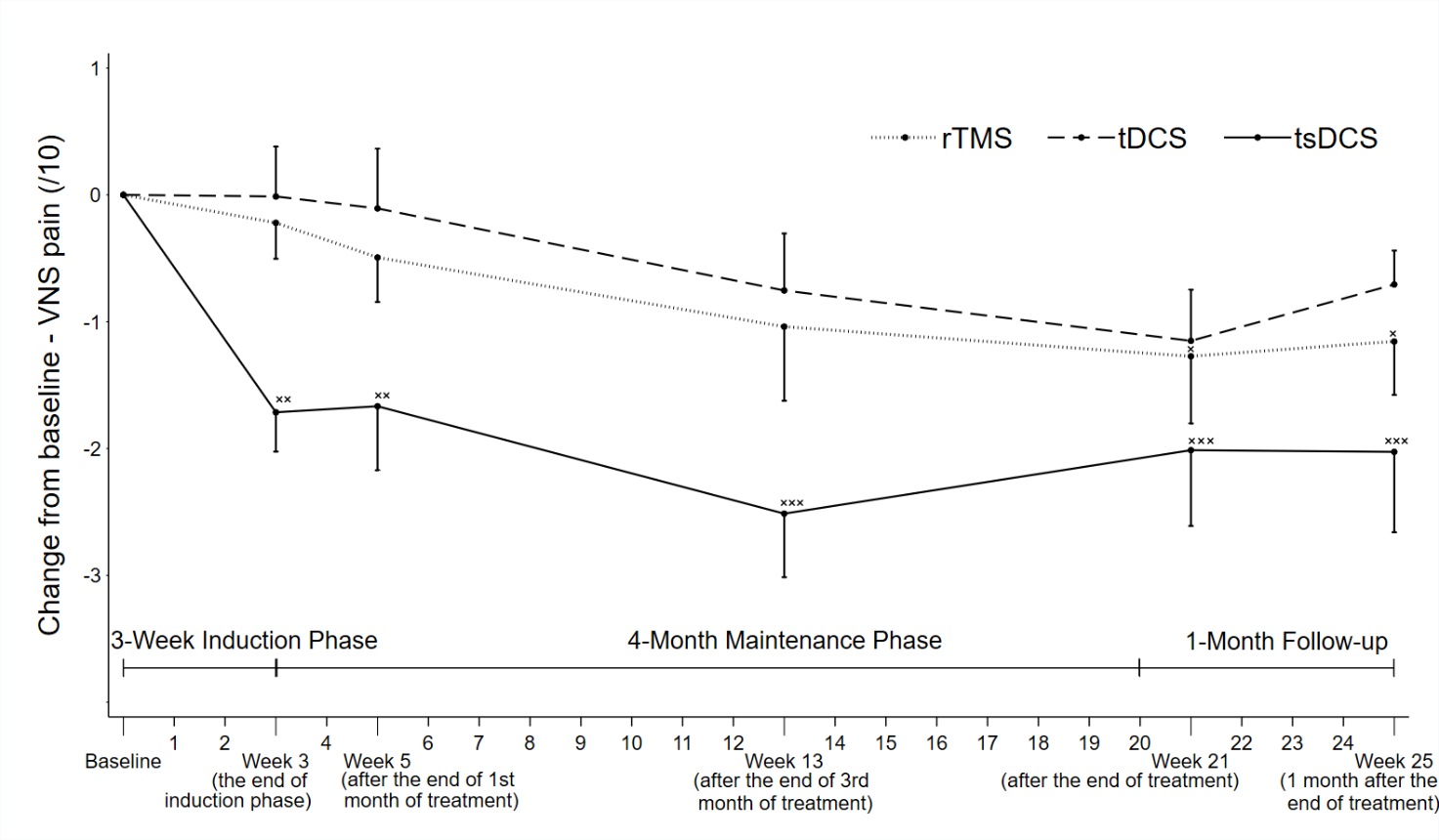


The line graph represents the mean values with standard errors bars in the per-protocol population. Changes from baseline in mean pain intensity rated on a 0-10 visual numeric scale (VNS) are plotted over the 25 weeks of follow-up for the three groups of patients with complex regional pain syndrome treated by high-frequency repetitive transcranial magnetic stimulation (rTMS), transcranial direct current stimulation (tDCS), or transcutaneous spinal direct current stimulation (tsDCS). Changes from baseline in mean VNS pain score significantly differed in the tsDCS group versus both rTMs and tDCS groups. Significant time-by-group interaction (P = 0.015, estimated by the use of two-way repeated measures analyses of variance model including stimulation group, time and the interaction of stimulation group with time) with Bonferroni’s post-hoc tests compared to baseline showing VNS pain score decrease in the tsDCS group at all time points and in the rTMS group at the last two time points (x p<0.05, xx p<0.01, xxx p<10-3).

# **Supplementary Table 1**

Primary outcome in Intention-to-Treat population: analysis of covariance (ANCOVA) used to compare groups at key time points, with baseline values used as covariates.

|  | **tsDCS** | **rTMS** | **tDCS** | **tsDCS vs rTMS** | **tsDCS vs tDCS** | **tDCS vs rTMS** |
| --- | --- | --- | --- | --- | --- | --- |
| Baseline | 6.6 (1.7) | 5.4 (1.8) | 6.3 (1.6) |  |  |  |
| Week 3, adjusted mean^#^ | 4.4 (3.8; 5.1) | 5.9 (5.1; 6.6) | 6.1 (5.4; 6.8) | -1.4 (-2.7; -0.2), P=0.019 | -1.7 (-2.9; -0.5), P=0.004 | 0.3 (-1.0; 1.5), P=0.999 |
| Week 5, adjusted mean^#^ | 4.6 (3.7; 5.5) | 5.4 (4.5; 6.3) | 6.1 (5.1; 7.0) | -0.8 (-2.5; 0.8), P=0.592 | -1.5 (-3.1; 0.1), P=0.077 | 0.6 (-1.0; 2.3), P=0.999 |
| Week 13, adjusted mean^#^ | 3.8 (2.8; 4.9) | 4.9 (3.8; 6.0) | 5.7 (4.6; 6.9) | -1.1 (-3.0; 0.9), P=0.521 | -1.9 (-3.8; 0.0), P=0.054 | 0.8 (-1.2; 2.8), P=0.922 |
| Week 21, adjusted mean | 4.3 (3.2; 5.4) | 4.7 (3.5; 5.8) | 5.4 (4.2; 6.5) | -0.4 (-2.3; 1.6), P=0.999 | -1.1 (-3.0; 0.9), P=0.523 | 0.7 (-1.3; 2.7), P=0.999 |
| Week 25, adjusted mean | 4.3 (3.3; 5.3) | 4.8 (3.7; 5.8) | 5.8 (4.7; 6.8) | -0.5 (-2.2; 1.3), P=0.999 | -1.5 (-3.2; 0.3), P=0.126 | 1.0 (-0.8; 2.8), P=0.507 |

Data are expressed as mean ± SD or mean with 95%CI. Adjusted means from baseline are provided with ANCOVA.

# Pairwise comparisons were analyzed by Bonferroni’s post hoc tests (95% CI and P) if P of difference between the three groups were ≤0.100.

# **Supplementary Table 2**

Secondary outcomes in Intention-to-Treat population: analysis of covariance (ANCOVA) used to compare groups at key time points, with baseline values used as covariates.

|  | **tsDCS** | **rTMS** | **tDCS** | **tsDCS vs rTMS** | **tsDCS vs tDCS** | **tDCS vs rTMS** |
| --- | --- | --- | --- | --- | --- | --- |
| **SF-12 Mental component scale** | | | |  |  |  |
| Baseline | 33.9 (8.0) | 40.9 (10.0) | 39.7 (14.2) |  |  |  |
| Day 90 adjusted mean^#^ | 44.1 (39.8; 48.4) | 36.5 (32.1; 40.9) | 39.9 (35.3; 44.4) | 7.6 (-0.1; 15.4), P=0.055 | 4.2 (-3.6; 12.0), P=0.546 | 3.4 (-4.4; 11.2), P=0.830 |
| Day 180 adjusted mean^#^ | 45.9 (39.9; 51.9) | 37.1 (30.9; 43.2) | 43.9 (37.5; 50.2) | 8.8 (-2.0; 19.6), P=0.143 | 2.0 (-8.9; 13.0), P=0.999 | 6.8 (-4.1; 17.7), P=0.378 |
| **SF-12 Physical component scale** | | | |  |  |  |
| Baseline | 31.2 (5.202) | 30.6 (4.9) | 28.3 (6.0) |  |  |  |
| Day 90 adjusted mean | 30.9 (28.8; 33.0) | 31.8 (29.6; 34.0) | 29.5 (27.1; 31.8) | -0.9 (-4.0; 2.1), P=0.547 | 1.4 (-1.8; 4.6), P=0.370 | -2.3 (-5.6; 0.9), P=0.152 |
| Day 180 adjusted mean | 32.6 (29.1; 36.1) | 33.6 (30.0; 37.3) | 30.6 (26.7; 34.5) | -1.1 (-6.1; 4.0), P=0.669 | 2.0 (-3.3; 7.3), P=0.452 | -3.0 (-8.4; 2.3), P=0.255 |
| **HADS-anxiety** | | | |  |  |  |
| Baseline | 12.3 (4.8) | 10.0 (3.6) | 11.5 (5.2) |  |  |  |
| Day 90 adjusted mean^#^ | 9.2 (7.8; 10.6) | 11.5 (10.0; 13.0) | 9.3 (7.7; 10.8) | -2.2 (-4.8; 0.3), P=0.101 | 0.0 (-2.6; 2.5), P=0.999 | -2.0 (-4.9; 0.4), P=0.123 |
| Day 180 adjusted mean | 8.0 (6.1; 9.9) | 10.0 (8.1; 12.0) | 8.5 (6.5; 10.5) | -2.0 (-4.7; 0.7), P=0.139 | -0.5 (-3.2; 2.7), P=0.720 | -1.5 (-4.3; 1.3), P=0.272 |
| **HADS-depression** | | | |  |  |  |
| Baseline | 10.7 (4.5) | 8.4 (3.4) | 9.3 (5.9) |  |  |  |
| Day 90 adjusted mean^#^ | 7.2 (5.7; 8.6) | 9.2 (7.7; 10.8) | 9.5 (8.0; 11.1) | -2.1 (-4.7; 0.5), P=0.155 | -2.4 (-5.0; 0.3), P=0.089 | 0.3 (-2.4; 3.0), P=0.999 |
| Day 180 adjusted mean | 6.8 (5.0; 8.5) | 9.0 (7.2; 10.9) | 8.5 (6.6; 10.4) | -2.3 (-4.8; 0.3), P=0.085 | -1.8 (-4.4; 0.9), P=0.180 | -0.5 (-3.2; 2.2), P=0.702 |
| **Q-DASH/WOMAC** | | | |  |  |  |
| Baseline | 69.6 (14.4) | 63.1 (15.8) | 62.4 (14.8) |  |  |  |
| Day 90 adjusted mean | 57.7 (49.3; 66.1) | 62.6 (53.9; 71.2) | 64.6 (55.6; 73.7) | -4.8 (-17.0; 7.3), P=0.419 | -6.9 (-19.4; 5.5), P=0.264 | 2.1 (-10.4; 14.5), P=0.735 |
| Day 180 adjusted mean | 55.9 (47.1; 64.6) | 60.2 (51.2; 69.2) | 62.1 (52.6; 71.6) | -4.4 (-17.0; 8.3), P=0.485 | -6.2 (-19.2; 6.8), P=0.335 | 1.9 (-11.1; 14.9), P=0.772 |
| **NPSI Total** | | | |  |  |  |
| Baseline | 54.6 (21.4) | 38.8 (15.8) | 51.3 (26.0) |  |  |  |
| Day 90 adjusted mean | 37.0 (27.8; 46.3) | 40.9 (31.1; 50.7) | 41.9 (31.9; 51.9) | -3.8 (-17.6; 10.0), P=0.574 | -4.8 (-18.3; 8.7), P=0.471 | 1.0 (-13.2; 15.1), P=0.887 |
| Day 180 adjusted mean | 29.7 (21.4; 38.1) | 33.4 (24.5; 42.2) | 38.4 (29.4; 47.4) | -3.6 (-16.1; 8.9), P=0.558 | -8.7 (-20.8; 3.5), P=0.157 | 5.0 (-7.7; 17.8), P=0.426 |

Data are expressed as mean ± SD or mean with 95%CI. Adjusted means from baseline are provided with ANCOVA.

# Pairwise comparisons were analyzed by Bonferroni’s post hoc tests (95% CI and P) if P of difference between the three groups were ≤0.100.

# **Supplementary Table 3**

Primary outcome: visual numeric pain scale in Per-Protocol population.

**F_Time×Stimulation 50,676_ = 1.51; P = 0.015**

|  | **tsDCS**  **(n = 12)** | **rTMS**  **(n = 11)** | **tDCS (n = 10)** | **tsDCS vs rTMS** | **tsDCS vs tDCS** | **tDCS vs rTMS** |
| --- | --- | --- | --- | --- | --- | --- |
| Baseline | 6.6 (1.7), n=12 | 5.4 (1.8), n=11 | 6.3 (1.6), n=10 |  |  |  |
| Week 3 unadjusted mean | 4.9 (2.1), n=12 | 5.1 (2.1), n=11 | 6.3 (1.5), n=10 |  |  |  |
| Week 3 absolute change^#^ | -1.7 (1.1), d=-1.6, P=0.001 | -0.2 (0.9), d=-0.2, P=0.999 | 0.0 (1.2), d=0.0, P=0.999 | -1.5 (-2.6; -0.3), d=-1.5, P=0.009 | -1.7 (-2.9; -0.5), d=-1.5, P=0.009 | 0.2 (-1.0; 1.4), d=0.2, P=0.999 |
| Week 3, adjusted mean^#^ | 4.4 (3.8; 5.1) | 5.8 (5.1; 6.6) | 6.1 (5.4; 6.8) | -1.4 (-2.7; -0.2), P=0.019 | -1.7 (-2.9; -0.5), P=0.004 | 0.3 (-1.0; 1.5), P=0.609 |
| Week 5 unadjusted mean | 5.0 (2.0), n=12 | 4.9 (1.8), n=11 | 6.2 (2.0), n=10 |  |  |  |
| Week 5 absolute change^#^ | -1.7 (1.8), d=-1.0, P=0.002 | -0.5 (1.2), d=-0.4, P=0.999 | -0.1 (1.5), d=-0.1, P=0.999 | -1.2 (-2.8; 0.4), d=-0.8, P=0.210 | -1.6 (-3.2; -0.1), d=-1.0, P=0.063 | 0.4 (-1.3; 2.0), d=0.3, P=0.999 |
| Week 5, adjusted mean^#^ | 4.6 (3.7; 5.5) | 5.4 (4.5; 6.3) | 6.1 (5.1; 7.0) | -0.8 (-2.5; 0.8), P=0.592 | -1.5 (-3.1; 0.1), P=0.077 | 0.6 (-1.0; 2.3), P=0.999 |
| Week 13 unadjusted mean | 4.1 (2.4), n=11 | 4.3 (1.9), n=11 | 5.6 (2.3), n=9 |  |  |  |
| Week 13 absolute change^#^ | -2.5 (1.7), d=-1.5, P=0.001 | -1.0 (1.9), d=-0.5, P=0.079 | -0.8 (1.4), d=-0.6, P=0.999 | -1.5 (-3.3; 0.4), d=-0.8, P=0.150 | -1.8 (-3.7; 0.2), d=-1.2, P=0.084 | 0.3 (-1.3; 2.2), d=0.2, P=0.909 |
| Week 13, adjusted mean^#^ | 3.7 (2.6; 4.8) | 4.9 (3.8; 6.0) | 5.4 (4.2; 6.6) | -1.2 (-3.2; 0.7), P=0.337 | -1.7 (-3.6; 0.2), P=0.098 | 0.5 (-1.5; 2.5), P=0.999 |
| Week 21, unadjusted mean | 4.6 (2.6), n=11 | 4.1 (1.8), n=11 | 5.2 (2.2), n=9 |  |  |  |
| Week 21 absolute change | -2.0 (2.0), d=-1.0, P<10-3 | -1.3 (1.8), d=-0.7, P=0.010 | -1.2 (1.2), d=-0.9, P=0.471 | -0.7 (-2.6; 1.1), d=-0.4, P=0.956 | -0.9 (-2.8; 1.1), d=-0.5, P=0.814 | 0.1 (-1.3; 2.1), d=0.1, P=0.999 |
| Week 21, adjusted mean | 4.2 (3.1; 5.3) | 4.7 (3.6; 5.8) | 5.0 (3.8; 6.2) | -0.5 (-2.4; 1.5), P=0.999 | -0.8 (-2.8; 1.1), P=0.900 | 0.3 (-1.7; 2.3), P=0.999 |
| Week 25, unadjusted mean | 4.6 (2.5), n=11 | 4.2 (2.0), n=11 | 5.7 (1.3), n=9 |  |  |  |
| Week 25 absolute change | -2.0 (2.1), d=-1.0, P<10-3 | -1.2 (1.4), d=-0.8, P=0.029 | -0.7 (0.8), d=-0.9, P=0.999 | -0.9 (-2.6; 0.8), d=-0.5, P=0.611 | -1.3 (-3.1; 0.5), d=-0.8, P=0.215 | 0.4 (-1.3; 2.2), d=0.4, P=0.999 |
| Week 25, adjusted mean | 4.2 (3.2; 5.2) | 4.8 (3.8; 5.8) | 5.5 (4.4; 6.5) | -0.6 (-2.4; 1.2), P=0.999 | -1.3 (-3.0; 0.5), P=0.243 | 0.7 (-1.1; 2.5), P=0.999 |

Data are expressed as mean ± SD or mean with 95%IC. Adjusted means from baseline are provided with ANCOVA. A negative change within groups means improvement. Between-group differences were calculated as tsDCS group results: a negative difference favors the tsDCS group. Comparisons to baseline were performed from Bonferroni’s post-hoc tests. Effect size is based on Cohen’s d. # Pairwise comparisons were analyzed by Bonferroni’s post hoc tests (95% CI and P) if P of difference between the three groups were ≤0.100

# **Supplementary Table 4**

Secondary outcomes in Per-Protocol population.

|  | **tsDCS (n = 12)** | **rTMS (n = 11)** | **tDCS (n = 10)** | **tsDCS vs rTMS** | **tsDCS vs tDCS** | **tsDCS vs rTMS** |
| --- | --- | --- | --- | --- | --- | --- |
| **SF-12 Mental component scale** (F_Time×Stimulation 4,50_ = 2.80; P = 0.042) | | | |  |  |  |
| BL | 33.9 (8.0), n=12 | 40.9 (10.0), n=11 | 39.7 (14.2), n=10 |  |  |  |
| D90 to BL^#^ | 8.1 (9.3), d=0.9, P=0.032, n=10 | -2.2 (5.1), d=-0.4, P=0.999, n=10 | 2.0 (7.5), d=0.3, P=0.999, n=8 | 10.3 (1.7; 18.9), d=1.3, P=0.015 | 6.1 (-3.0; 15.2), d=0.7, P=0.294 | 4.2 (-4.9; 13.3), d=0.7, P=0.744 |
| D90 adj. mean^#^ | 46.0 (41.1; 50.8) | 37.2 (32.4; 42.1) | 40.6 (35.4; 45.9) | 8.7 (-0.1; 17.4), P=0.048 | 5.3 (-3.6; 14.2), P=0.413 | 3.4 (-5.5; 12.3), P=0.999 |
| D180 to BL^#^ | 9.0 (9.9), d=0.9, P=0.032, n=11 | -1.2 (9.3), d=-0.1, P=0.999, n=11 | 9.0 (12.2), d=0.7, P=0.194, n=6 | 10.2 (-0.9; 21.3), d=1.1, P=0.081 | 0.0 (-13.2; 13.2), d=0.0, P=0.999 | 10.2 (-3.1; 23.4), d=1.0, P=0.179 |
| D180 adj. mean^#^ | 46.3 (39.7; 52.9) | 36.6 (30.0; 43.2) | 46.5 (37.8; 55.2) | 9.7 (-2.2; 21.6), P=0.138 | -0.2 (-13.8; 13.4), P=0.999 | 9.9 (-3.8; 23.6), P=0.225 |
| **SF-12 Physical component scale** (F_Time×Stimulation 4,50_ = 0.42; P = 0.762) | | | |  |  |  |
| BL | 31.2 (5.2), n=12 | 30.6 (4.9), n=11 | 28.3 (6.0), n=10 |  |  |  |
| D90 to BL | 0.6 (4.7), d=0.1, P=0.694, n=10 | 1.7 (3.7), d=0.5, P=0.182, n=10 | -0.3 (3.9), d=-0.1, P=0.859, n=8 | -1.2 (-5.1; 2.9), d=-0.3, P=0.567 | 0.9 (-3.5; 5.2), d=0.2, P=0.684 | -2.0 (-5.7; 1.8), d=-0.5, P=0.292 |
| D90 adj. mean | 31.0 (28.5; 33.5) | 32.0 (29.5; 34.5) | 28.9 (26.0; 31.8) | -1.1 (-4.6; 2.5), P=0.537 | 2.1 (-1.8; 6.0), P=0.280 | -3.1 (-7.0; 0.7), P=0.107 |
| D180 to BL | 2.7 (5.5), d=0.5, P=0.132, n=11 | 3.6 (7.8), d=0.5, P=0.163, n=11 | 0.3 (4.3), d=0.1, P=0.856, n=6 | -0.8 (-6.8; 5.2), d=-0.1, P=0.780 | 2.4 (-3.2; 7.9), d=0.5, P=0.373 | -3.2 (-10.6; 4.2), d=-0.5, P=0.369 |
| D180 adj. mean | 32.5 (28.4; 36.6) | 33.4 (29.3; 37.4) | 30.4 (24.6; 36.2) | -0.8 (-6.5; 4.8), P=0.762 | 2.1 (-5.2; 9.3), P=0.558 | -2.9 (-10.1, 4.2), P=0.407 |
| **HADS-anxiety** (F_Time×Stimulation 4,51_ = 2.52; P = 0.066) | | | |  |  |  |
| BL | 12.3 (4.8), n=12 | 10.0 (3.6), n=11 | 11.5 (5.2), n=10 |  |  |  |
| D90 to BL^#^ | -2.4 (3.2), d=-0.8, P=0.047, n=10 | 0.2 (1.7), d=0.1, P=0.999, n=10 | -2.5 (1.9), d=-1.3, P=0.047, n=8 | -2.6 (-5.3; 0.1), d=-1.0, P=0.066 | 0.1 (-2.8; 3.0), d=0.0, P=0.999 | -2.7 (-5.6; 0.2), d=-1.5, P=0.074 |
| D90 adj. mean^#^ | 8.5 (6.9; 10.1) | 11.1 (9.5; 12.7) | 8.4 (6.6; 10.2) | -2.6 (-5.4; 0.2), P=0.078 | 0.1 (-2.9; 3.1), P=0.999 | -2.7 (-5.7; 0.3), P=0.089 |
| D180 to BL | -3.7 (4.1), d=-0.9, P=0.004, n=11 | -1.0 (2.0), d=-0.5, P=0.279, n=11 | -3.6 (3.2), d=-1.1, P=0.007, n=7 | -2.7 (-5.6; 0.1), d=-0.8, P=0.061 | -0.2 (-4.0; 3.7), d=0.0, P=0.933 | -2.6 (-5.1; 0.1), d=-1.0, P=0.059 |
| D180 adj. mean | 7.7 (5.7; 9.6) | 10.0 (8.0; 11.9) | 8.0 (5.6; 10.4) | -2.3 (-5.1; 0.4), P=0.096 | -0.4 (-3.4; 2.7), P=0.814 | -2.0 (-5.1; 1.2), P=0.213 |
| **HADS-depression** (F_Time×Stimulation 4,51_ = 2.17; P = 0.086) | | | |  |  |  |
| BL | 10.7 (4.5), n=12 | 8.4 (3.4), n=11 | 9.3 (5.9), n=10 |  |  |  |
| D90 to BL^#^ | -2.9 (2.9), d=-1.0, P=0.005, n=10 | -0.3 (2.1), d=-0.1, P=0.999, n=10 | 0.0 (2.3), d=0.0, P=0.999, n=8 | -2.6 (-5.5; 0.3), d=-1.0, P=0.084 | -2.9 (-5.9; 0.1), d=-1.1, P=0.064 | 0.3 (-2.7; 3.3), d=0.1, P=0.999 |
| D90 adj. mean^#^ | 6.5 (4.8; 8.1) | 9.0 (7.3; 10.6) | 9.3 (7.5; 11.2) | -2.5 (-5.4; 0.5), P=0.122 | -2.9 (-5.9; 0.2), P=0.077 | 0.4 (-2.7; 3.5), P=0.999 |
| D180 to BL | -3.1 (2.5), d=-1.3, P=0.002, n=11 | -0.45 (3.9), d=-0.1, P=0.999, n=11 | -1.1 (2.3), d=-0.5, P=0.585, n=7 | -2.6 (-5.5; 0.3), d=-0.8, P=0.073 | -1.9 (-4.4; 0.5), d=-0.8, P=0.112 | -0.7 (-4.2; 2.8), d=-0.2, P=0.680 |
| D180 adj. mean | 6.7 (4.7; 8.6) | 9.1 (7.1; 11.0) | 8.6 (6.2; 11.1) | -2.4 (-5.2; 0.4), P=0.084 | -2.0 (-5.0; 1.1), P=0.199 | -0.5 (-3.6; 2.7), P=0.772 |
| **Q-DASH/WOMAC** (F_Time×Stimulation 4,49_ = 0.74; P = 0.570) | | | |  |  |  |
| BL | 69.6 (14.4), n=12 | 63.1 (15.8), n=11 | 62.4 (14.8), n=10 |  |  |  |
| D90 to BL | -9.9 (16.8), d=-0.6, P=0.133, n=10 | -2.6 (13.3), d=-0.2, P=0.999, n=10 | -0.2 (14.7), d=0.0, P=0.999, n=8 | -7.3 (-21.5; 6.9), d=-0.5, P=0.295 | -9.7 (-25.7; 6.3), d=-0.6, P=0.216 | 2.4 (-11.6; 16.4), d=-0.2, P=0.721 |
| D90 adj. mean | 55.6 (46.0; 65.2) | 60.8 (51.1; 70.5) | 65.3 (54.6; 76.0) | -5.2 (-19.0; 8.6), P=0.446 | -9.7 (-24.0; 4.7), P=0.177 | 4.5 (-10.1; 19.1), P=0.533 |
| D180 to BL | -10.9 (14.3), d=-0.8, P=0.062, n=11 | -7.2 (16.6), d=-0.4, P=0.529, n=9 | 0.3 (10.8), d=0.0, P=0.999, n=7 | -3.7 (-18.2; 10.9), d=-0.2, P=0.602 | -11.2 (-24.7; 2.2), d=-0.9, P=0.096 | 7.6 (-8.0; 23.1), d=0.5, P=0.315 |
| D180 adj. mean | 55.1 (46.1; 64.2) | 58.2 (48.2; 68.2) | 66.0 (54.6; 77.3) | -3.1 (-16.6; 10.4), P=0.640 | -10.8 (-25.3; 3.6), P=0.135 | 7.8 (-7.3; 22.8), P=0.299 |
| **NPSI Total** (F_Time×Stimulation 4,49_ = 0.69; P = 0.604) | | | |  |  |  |
| BL | 54.6 (21.4), n=12 | 38.7 (16.6), n=10 | 51.3 (26.0), n=10 |  |  |  |
| D90 to BL | -16.0 (21.8), d=-0.7, P=0.028, n=9 | -7.8 (10.8), d=-0.7, P=0.110, n=9 | -8.5 (14.7), d=-0.6, P=0.472, n=8 | -8.2 (-25.4; 9.0), d=-0.5, P=0.326 | -7.5 (-27.0; 12.0), d=-0.4, P=0.425 | -0.7 (-14.0; 12.5), d=-0.1, P=0.909 |
| D90 adj. mean | 32.9 (21.4; 44.4) | 38.3 (26.4; 50.3) | 40.6 (28.4; 52.9) | -5.4 (-22.4; 11.5), P=0.513 | -7.7 (-24.3; 8.9), P=0.344 | 2.3 (-15.3; 19.9), P=0.791 |
| D180 to BL | -21.8 (16.1), d=-1.4, P=0.002, n=11 | -13.4 (12.3), d=-1.1, P=0.003, n=10 | -12.1 (15.9), d=-0.8, P=0.143, n=7 | -8.4 (-21.6; 4.8), d=-0.6, P=0.197 | -9.7 (-26.1; 6.7), d=-0.6, P=0.230 | 1.3 (-13.3; 15.8), d=0.1, P=0.856 |
| D180 adj. mean | 28.6 (20.0; 37.2) | 32.4 (23.0; 41.7) | 39.4 (28.5, 50.2) | -3.8 (-16.7; 9.2), P=0.554 | -10.7 (-24.4; 2.9), P=0.117 | 7.0 (-7.8; 21.7), P=0.339 |

Abbreviations: *BL*, baseline, visit 2; *D90,* visit 3, end of 3^rd^ month of treatment; *D180*, visit 4, 1 month after the end of treatment; *d*, Cohen’s d. effect size; *SF-12*, 12-item Short-Form Health Survey questionnaire (0-100): Physical and Mental subscales; *HADS*, hospital anxiety and depression scale (0-21): Anxiety and Depression subscales; *Q-DASH*, Quick Disabilities of Arm, Shoulder and Hand scale normalized to 100 (score range 0-100); *WOMAC*, Western Ontario and McMaster Universities Osteoarthritis Index for knee and hip (normalized 0-100); *ESC*, electrochemical sweat conductance obtained with SUDOSCAN^®^.

Data are expressed as absolute change from baseline (mean ± SD) and adjusted mean for baseline values (95%IC) from ANCOVA. Comparisons to baseline were performed from Bonferroni’s post-hoc tests. ^#^ Pairwise comparisons were analyzed by Bonferroni’s post hoc tests (95% CI and P) if P of difference between the three groups were ≤0.100. Effect size is based on Cohen’s d.

# **Supplementary Table 5**

Primary outcome: visual numeric pain scale in Intention-to-Treat population, tDCS vs rTMs

|  | **rTMS (n = 11)** | **tDCS (n = 10)** | **tDCS vs rTMS** |
| --- | --- | --- | --- |
| Baseline | 5.4 (1.8) | 6.3 (1.6) |  |
| Week 3, absolute change^#^ | -0.2 (0.9) | 0.0 (1.2) | 0.2 (1.0; 1.4), d=0.2, P=0.999 |
| Week 3, adjusted mean^#^ | 5.9 (5.1; 6.6) | 6.1 (5.4; 6.8) | 0.3 (-1.0; 1.5), P=0.999 |
| Week 5, absolute change^#^ | -0.5 (1.2) | -0.1 (1.5) | 0.4 (-1.3; 2.0), d=0.3, P=0.999 |
| Week 5, adjusted mean^#^ | 5.4 (4.5; 6.3) | 6.1 (5.1; 7.0) | 0.6 (-1.0; 2.3), P=0.999 |
| Week 13, absolute change^#^ | -1.0 (1.9) | -0.4 (1.7) | 0.6 (-1.3; 2.6), d=0.3, P=0.999 |
| Week 13, adjusted mean^#^ | 4.9 (3.8; 6.0) | 5.7 (4.6; 6.9) | 0.8 (-1.2; 2.8), P=0.922 |
| Week 21, absolute change | -1.2 (1.4) | -0.4 (1.3) | 0.5 (-1.5; 2.5), d=0.3, P=0.999 |
| Week 21, adjusted mean | 4.7 (3.5; 5.8) | 5.4 (4.2; 6.5) | 0.7 (-1.3; 2.7), P=0.999 |
| Week 25, absolute change | -1.2 (1.4) | -0.4 (1.3) | 0.8 (-1.0; 2.6), d=0.6, P=0.852 |
| Week 25, adjusted mean | 4.8 (3.7; 5.8) | 5.8 (4.7; 6.8) | 1.0 (-0.8; 2.8), P=0.507 |

Abbreviations: 3-W, week 3 after half of the induction phase; 5-W, week 5 after the end of the induction phase; 13-W, week 13 after the end of the 3rd month of treatment; 21-W, week 21 after the end of the treatment; 25-W, week 25 1 month after the end of the treatment.

Data are expressed as mean ± SD or mean with 95%IC. Adjusted means for baseline differences are provided from ANCOVA. A negative change within groups means improvement. Between-group differences were calculated as tsDCS group results: a negative difference favors the tsDCS group. Effect size is based on Cohen’s d. # Pairwise comparisons were analyzed by Bonferroni’s post hoc tests (95% CI and P) if P of difference between the three groups were ≤0.100

# **Supplementary Table 6**

Secondary outcomes in Intention-to-Treat population, tDCS vs rTMs

|  | **rTMS (n = 11)** | **tDCS (n = 10)** | **tDCS vs rTMS** |
| --- | --- | --- | --- |
| **SF-12 Mental component scale** | | |  |
| BL | 40.9 (10.0) | 39.7 (14.2) |  |
| D90 to BL^#^ | -2.0 (4.9), d=-0.4 | 1.6 (6.6), d=0.2 | 3.6 (-4.3; 11.5), d=0.6, P=0.769 |
| D90 adj. mean^#^ | 36.5 (32.1; 40.9) | 39.9 (35.3; 44.4) | 3.4 (-4.4; 11.2), P=0.830 |
| D180 to BL^#^ | -1.2 (9.3), d=-0.1 | 5.7 (10.1), d=0.6 | 6.9 (-3.9; 17.6), d=0.7, P=0.346 |
| D180 adj. mean^#^ | 37.1 (30.9; 43.2) | 43.9 (37.5; 50.2) | 6.8 (-4.1; 17.7), P=0.378 |
| **SF-12 Physical component scale** | | |  |
| BL | 30.6 (4.9) | 28.3 (6.0) |  |
| D90 to BL | 1.5 (3.6), d=0.4 | -0.2 (3.4), d=-0.1 | -1.7 (-4.9; 1.4), d=-0.5, P=0.266 |
| D90 adj. mean | 31.8 (29.6; 34.0) | 29.5 (27.1; 31.8) | -2.3 (-5.6; 0.9), P=0.152 |
| D180 to BL | 3.5 (7.8), d=0.5 | 0.4 (3.2), d=0.1 | -3.1 (-8.7; 2.4), d=-0.5, P=0.252 |
| D180 adj. mean | 33.6 (30.0; 37.3) | 30.6 (26.7; 34.5) | -3.0 (-8.4; 2.3), P=0.255 |
| **HADS-anxiety** | | |  |
| BL | 10.0 (3.6) | 11.5 (5.2) |  |
| D90 to BL^#^ | 0.2 (1.6), d=0.1 | -2.0 (2.0), d=-1.0 | -2.2 (-4.8; 0.4), d=-1.2, P=0.119 |
| D90 adj. mean^#^ | 11.5 (10.0; 13.0) | 9.3 (7.7; 10.8) | -2.2 (-4.9; 0.4), P=0.123 |
| D180 to BL | -1.0 (2.0), d=-0.5 | -2.8 (3.0)^**^, d=-0.9 | -1.8 (-4.1; 0.5), d=-0.7, P=0.117 |
| D180 adj. mean | 10.0 (8.1; 12.0) | 8.5 (6.5; 10.5) | -1.5 (-4.3; 1.3), P=0.272 |
| **HADS-depression** | | |  |
| BL | 8.4 (3.4) | 9.3 (5.9) |  |
| D90 to BL^#^ | -0.3 (2.0), d=0.1 | 0.0 (2.1), d=0.0 | 0.3 (-2.4; 2.9), d=0.1, P=0.999 |
| D90 adj. mean^#^ | 9.2 (7.7; 10.8) | 9.5 (8.0; 11.1) | 0.3 (-2.4; 3.0), P=0.999 |
| D180 to BL | -0.45 (3.9), d=-0.1 | -1.0 (1.9), d=-0.5 | -0.5 (-3.4; 2.3), d=-0.2, P=0.695 |
| D180 adj. mean | 9.0 (7.2; 10.9) | 8.5 (6.6; 10.4) | -0.5 (-3.2; 2.2), P=0.702 |
| **Q-DASH/WOMAC** | | |  |
| BL | 63.1 (15.8) | 62.4 (14.8) |  |
| D90 to BL | -2.3 (12.6), d=-0.2 | -0.1 (13.0), d=0.0 | 2.2 (-9.5; 13.9), d=0.2, P=0.698 |
| D90 adj. mean | 40.9 (31.1; 50.7) | 41.9 (31.9; 51.9) | 2.1 (-10.4; 14.5), P=0.735 |
| D180 to BL | -4.7 (16.2), d=-0.3 | -2.7 (12.9), d=-0.2 | 2.0 (-11.5; 15.4), d=0.1, P=0.763 |
| D180 adj. mean | 33.4 (24.5; 42.2) | 38.4 (29.4; 47.4) | 1.9 (-11.1; 14.9), P=0.772 |
| **NPSI Total** | | |  |
| BL | 38.8 (15.8) | 51.3 (26.0) |  |
| D90 to BL | -6.4 (10.2), d=-0.6 | -6.8 (13.5), d=-0.5 | -0.4(-11.3; 10.4), d=0.0, P=0.934 |
| D90 adj. mean | 62.6 (53.9; 71.2) | 64.6 (55.6; 73.7) | 1.0 (-13.2; 15.1), P=0.887 |
| D180 to BL | -12.8 (11.8)^**^, d=-1.1 | -10.6 (14.4=, d=-0.7 | 2.2 (-9.8; 14.2), d=0.2, P=0.703 |
| D180 adj. mean | 60.2 (51.2; 69.2) | 62.1 (52.6; 71.6) | 5.0 (-7.7; 17.8), P=0.426 |

Abbreviations: *BL*, baseline, visit 2; *D90,* visit 3, end of 3^rd^ month of treatment; *D180*, visit 4, 1 month after the end of treatment; *d*, Cohen’s d. effect size; *SF-12*, 12-item Short-Form Health Survey questionnaire (0-100): Physical and Mental subscales; *HADS*, hospital anxiety and depression scale (0-21): Anxiety and Depression subscales; *Q-DASH*, Quick Disabilities of Arm, Shoulder and Hand scale normalized to 100 (score range 0-100); *WOMAC*, Western Ontario and McMaster Universities Osteoarthritis Index for knee and hip (normalized 0-100); *ESC*, electrochemical sweat conductance obtained with SUDOSCAN^®^. Data are expressed as absolute change from baseline (mean ± SD) and adjusted mean for baseline values (95%IC) from ANCOVA. Effect size is based on Cohen’s d. ^#^ Pairwise comparisons were analyzed by Bonferroni’s post hoc tests (95% CI and P) if P of difference between the three groups were ≤0.100. Bonferroni’s post-hoc test compared to baseline: * P<0.05; ** P<0.01; *** P<0.001
